# Early girl is a novel component of the Fat signaling pathway

**DOI:** 10.1371/journal.pgen.1007955

**Published:** 2019-01-30

**Authors:** Jyoti R. Misra, Kenneth D. Irvine

**Affiliations:** Waksman Institute and Department of Molecular Biology and Biochemistry, Rutgers University, Piscataway, New Jersey; New York University, UNITED STATES

## Abstract

The *Drosophila* protocadherins Dachsous and Fat regulate growth and tissue polarity by modulating the levels, membrane localization and polarity of the atypical myosin Dachs. Localization to the apical junctional membrane is critical for Dachs function, and the adapter protein Vamana/Dlish and palmitoyl transferase Approximated are required for Dachs membrane localization. However, how Dachs levels are regulated is poorly understood. Here we identify the *early girl* gene as playing an essential role in Fat signaling by limiting the levels of Dachs protein. *early girl* mutants display overgrowth of the wings and reduced cross vein spacing, hallmark features of mutations affecting Fat signaling. Genetic experiments reveal that it functions in parallel with Fat to regulate Dachs. *early girl* encodes an E3 ubiquitin ligase, physically interacts with Dachs, and regulates its protein stability. Concomitant loss of *early girl* and *approximated* results in accumulation of Dachs and Vamana in cytoplasmic punctae, suggesting that it also regulates their trafficking to the apical membrane. Our findings establish a crucial role for *early girl* in Fat signaling, involving regulation of Dachs and Vamana, two key downstream effectors of this pathway.

## Author summary

During development, organs grow to achieve a consistent final size. The evolutionarily conserved Hippo signaling network plays a central role in organ size control, and when dysregulated can be associated with cancer and other diseases. Fat signaling is one of several upstream pathways that impinge on Hippo signaling to regulate organ growth. We describe here identification of the *Drosophila early girl* gene as a new component of the Fat signaling pathway. We show that Early girl controls Fat signaling by regulating the levels of the Dachs protein. However Early girl differs from other Fat signaling regulators in that it doesn’t influence planar cell polarity or control the polarity of Dachs localization. *early girl* encodes a conserved protein that is predicted to influence protein stability, and it can physically associate with Dachs. We also discovered that Early girl acts together with another protein, called Approximated, to regulate the sub-cellular localization of Dachs and a Dachs-interacting protein called Vamana. Altogether, our observations establish Early girl as an essential component of Fat signaling that acts to regulate the levels and localization of Dachs and Vamana.

## Introduction

Precise coordination of growth and morphogenesis during development is critical for formation of organs of correct proportions and optimal function. This is achieved through the cumulative effect of biochemical signaling through morphogens and biomechanical signals mediated through the actomyosin network. The protocadherins Dachsous (Ds) and Fat initiate a signaling cascade (Fat signaling), which functions to restrict growth by activating the Hippo signaling pathway [1, 2] and influences morphogenesis by modulating planar cell polarity (PCP) [3–6]. Fat signaling regulates the Hippo pathway and influences PCP by modulating membrane localization of the atypical myosin Dachs. Multiple studies have provided insight both into the mechanisms by which Dachs influences the Hippo pathway and PCP, and into how Fat regulates Dachs [7–18]. However, the mechanisms that control Dachs levels and membrane localization are still not completely understood.

Ds and Fat are protocadherins with large extracellular domains (ECD) and small intracellular domains (ICD) and localize to the apico-lateral membrane just apical to the adherens junctions. The ECDs of Ds and Fat interact with each other across the cell-cell junctions in a heterophilic manner and the Golgi resident kinase, Four-jointed (Fj), modulates this interaction by phosphorylating their ECDs [19–22]. In most developing tissues Ds and Fj are expressed in opposite gradients under the influence of morphogens [2]. The heterophilic interaction between Ds and Fat and the graded expression of Ds and Fj contributes to planar polarized localization of Ds and Fat within cells, where in the developing *Drosophila* wing Fat is preferentially enriched on the proximal side while Ds is enriched on the distal side [23–25]. Ds and Fat then regulate the levels and polarity of Dachs at cell membranes to influence Hippo signaling and PCP. In absence of Fat, increased amounts of Dachs accumulate around the entire circumference of cells [8, 24]. Conversely, overexpression of Fat or just the Fat ICD displaces Dachs from the membrane into the cytoplasm [8, 16].

The Hippo signaling pathway plays a central role in growth regulation and includes Hippo and its cofactor Salvador and Warts (Wts) and the Warts cofactor Mats [26]. Hippo phosphorylates and activates Wts, which in turn phosphorylates the transcriptional co-activator Yorkie (Yki). Phosphorylated Yki is sequestered in the cytoplasm. When the pathway is inactive, unphosphorylated Yki translocates into the nucleus, where it associates with Scalloped and induces the transcription of downstream target genes such as *expanded (ex)*, *bantam* (*ban*), and *cyclin E*, which modulate Hippo pathway activity, stimulate growth, and regulate cell fate. Multiple upstream regulators impinge on Wts to regulate Hippo signaling, including Fat signaling. Fat affects the membrane localization of Expanded, the levels of Wts and the interaction of Wts with Mats [7, 11, 27–30]. Dachs is required for each of these effects on Hippo signaling. Similarly, Fat signaling influences PCP signaling partly by regulating the Spiny legs isoform of the *prickle* locus [12, 13] and partly through an influence on junctional tension [31], and Dachs is involved in both of these processes.

Dachs localizes to the apico-lateral plasma membrane in a planar polarized manner [8]. In the developing wing discs Dachs localizes to the distal side of the cell [8, 23, 24, 32]. Several proteins that influence membrane localization of Dachs have been identified as components of the Fat signaling pathway. *vamana (vam/Dlish)* encodes an adapter protein with three SH3 domains that physically connects Dachs with the Fat and Ds ICDs, interacting with Dachs through its second SH3 domain and interacting with the Fat and Ds ICD through its first and third SH3 domains [14, 15]. Vam physically interacts with the C-terminal region of Dachs. Like Dachs, Vam localizes to the apical plasma membrane with preferential enrichment on the distal side of wing cells. In absence of Vam, Dachs fails to localize to the membrane and accumulates in the cytoplasm. Approximated encodes a palmitoyl transferase and is also required for Dachs membrane localization [33]. Although, the exact mechanism is still not clear, it can physically interact with both Dachs and Vam and can palmitoylate Vam and the Fat ICD when overexpressed [15, 34].

Despite progress in our understanding of the Fat signaling pathway, how Dachs localizes to the apical membrane and how its levels at the membrane are regulated is still not completely understood. Here we report the isolation and characterization of the *early girl* (*elgi*) gene as a key regulator of Fat-Hippo signaling. Loss of *elgi* function promotes growth by up-regulating Yki activity. This arises from accumulation of high levels of Dachs and Vam at the apical plasma membrane. Elgi physically interacts with Dachs and regulates Dachs and Vam by controlling their protein levels. Further, we show that concomitant loss of Elgi and App prevents Dachs and Vam localization to the subapical membrane and results in their accumulation in cytoplasmic punctae, suggesting that Elgi and App coordinately regulate trafficking and membrane localization of Dachs and Vam. Under this condition, Dachs is primarily affected and Vam gets mislocalized due to its interaction with Dachs. Our observations establish Elgi as an important component of the Fat signaling pathway.

## Results

### *elgi* mutants display phenotypes similar to loss of *fat*

**Fig 1:**
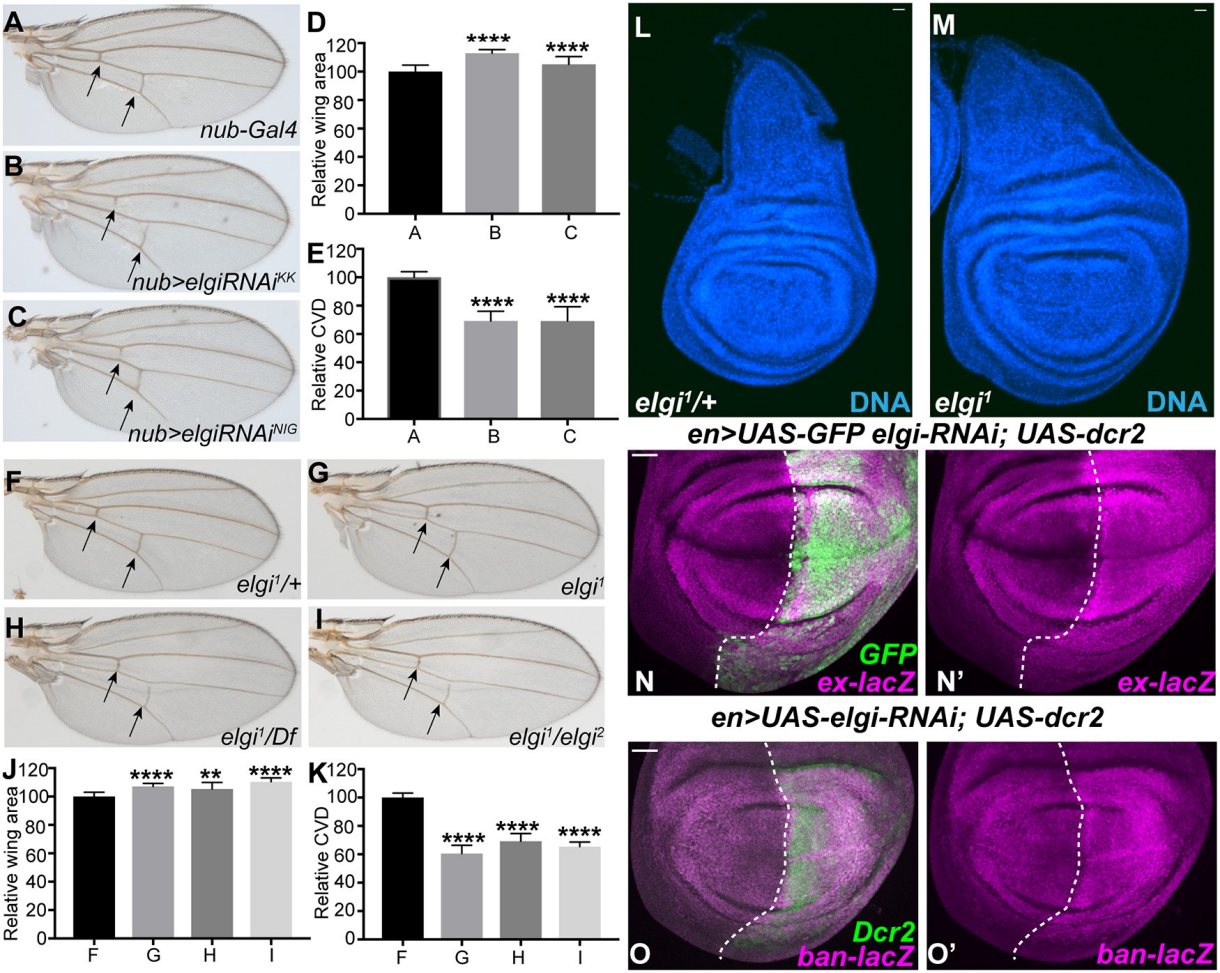
*elgi* mutant phenotypes. (A-C) Adult male wings from flies expressing *nub-Gal4 UAS-Dcr2* control (A), *nub-Gal4 UAS-Dcr2 UAS-elgi-RNAi*^*KK*^ (VDRC109617) (B), and *nub-Gal4 UAS-Dcr2 UAS-elgi-RNAi*^*NIG*^ (17033R-3) (C). Arrows point to the crossveins. (D) Histogram of relative wing areas in flies of the genotypes in panels A-C, as indicated (normalized to the average area of control wings); mean of B is 112.9, and C is 105.2. (E) Histogram of the ratio of crossvein distance (CVD) to wing length, (normalized to the average CVD/wing length in control wings) in flies of the genotypes in panels A-C, as indicated. (F-I) Adult male wings from *elgi*^*1*^*/+* (control) (F), homozygous *elgi*^*1*^ (G), hemizygous *elgi*^*1*^*/Df(3L) BSC575* (H), trans-heterozygous *elgi*^*1*^ / *elgi*^*2*^ (I) flies. Arrows point to the crossveins. (J) Histogram of relative wing areas (normalized to the average area of control wings) in flies of the genotypes in panels F-I, as indicated. Mean of G is 107.1; H is 105.4 and I is 110.4. (K) Histogram of the ratios of crossvein distance (CVD) to wing length, (normalized to the average CVD/wing length ratio of control wings) in flies of the genotypes in panels F-I, as indicated. Data are shown as mean ± SD from measurements of 10–13 wings per genotype. **P<0.002 and ****P<0.001, (Student’s *t* test between control and the other genotypes); (L,M) Wing imaginal discs from age matched control *elgi*^*1*^*/+* and homozygous *elgi*^*1*^ 4 day old third instar larvae. (N-O’) Third instar wing imaginal discs expressing *en-Gal4 UAS-dcr2 UAS-GFP UAS-elgi-RNAi* and *ex-lacZ* (N, N’) or *ban-lacZ* (O, O’) stained for expression of *ex-lacZ* or *ban-lacZ* (magenta), with posterior cells marked by expression of GFP or Dcr2. Dashed white line marks the A-P compartment boundary. Scale bar is 33 μm.

To identify contributions of protein stability to wing growth and patterning, we conducted a RNAi-based genetic screen, in which we depleted the RING domain E3 ubiquitin ligases encoded by the *Drosophila* genome within the wing pouch region of the developing wing imaginal disc using UAS RNAi lines and a *nub-Gal4* driver. From this screen, we identified *elgi*, based on an increase in size of the adult wing, and a reduced spacing between the anterior and posterior cross veins when it is knocked down by either of two independent RNAi lines (Fig 1A–1E). These phenotypes are hallmark features of mutations in components of the Fat signaling pathway [8, 14, 15, 33, 35–39], suggesting that *elgi* could participate in Fat signaling.

*elgi* was previously characterized for a role in oogenesis, and named for a mutant phenotype including premature meiotic maturation [40]. Loss of function alleles that were isolated based on this oogenesis phenotype, *elgi*^*1*^ and *elgi*^*2*^, also increase the size of adult wings and reduce spacing between the anterior and posterior cross veins when homozygous, or when hemizygous with a chromosomal deficiency that deletes the *elgi* locus (Fig 1F–1K). *elgi*^*1*^ mutant wing discs are also larger than control wing discs (Fig 1L and 1M). These observations confirm that *elgi* regulates wing growth and patterning.

Fat signaling regulates growth by controlling Hippo signaling. To examine the possibility that *elgi* regulates wing size through Hippo signaling, we depleted *elgi* in the posterior compartment of wing imaginal discs by expressing UAS-RNAi *elgi* under the control of *en-gal4*, and then examined the effect of this Elgi depletion on the levels of the Yki target genes *ex* and *ban*. Using *ex-lacZ* and *ban-lacZ* reporter genes, we observed that loss of *elgi* increased Yki activity (Fig 1N and 1O), consistent with the inference that the increased wing size observed is due to impairment of Fat-Hippo signaling.

### Elgi regulates Dachs and Vam levels

**Fig 2:**
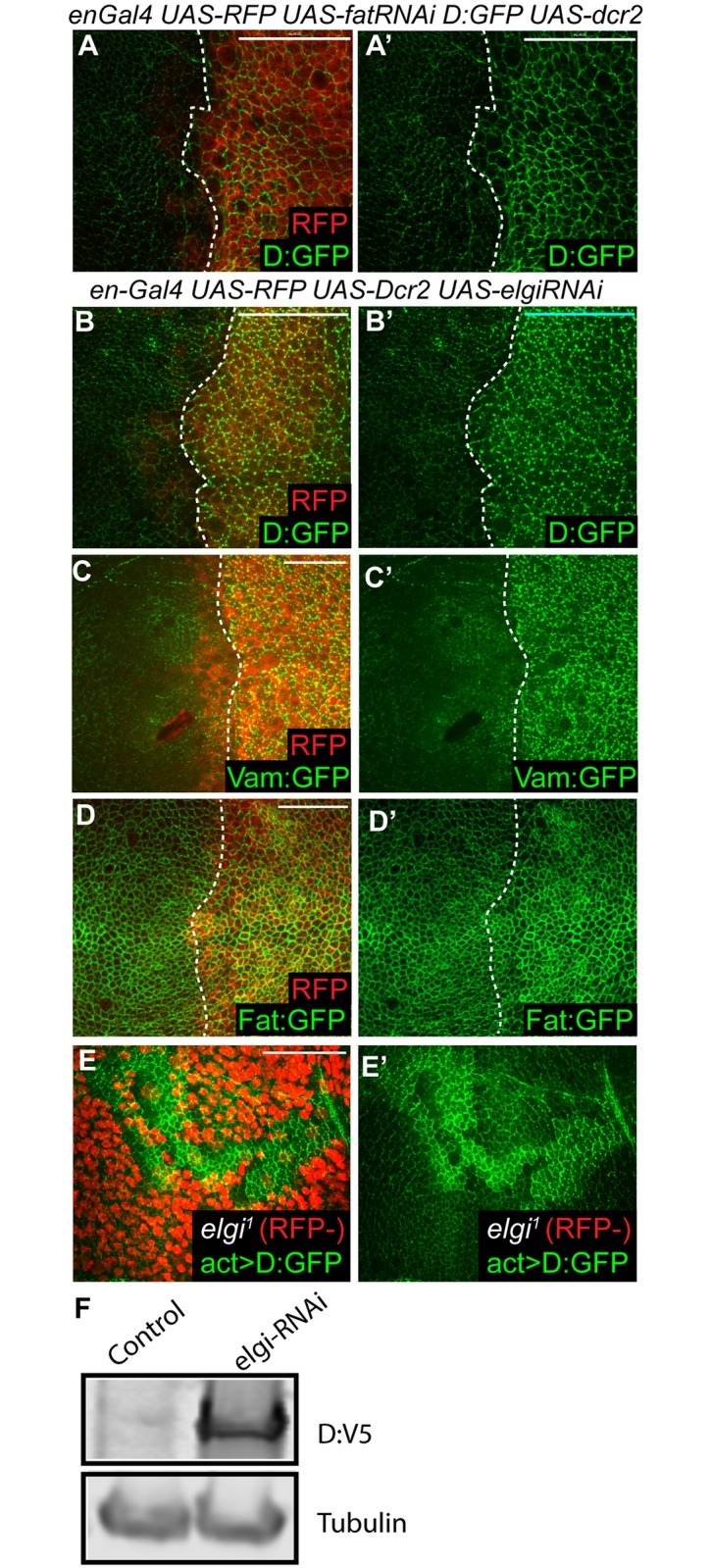
*elgi* regulates the levels of Dachs, Vam and Fat. (A-D) Horizontal apical sections of wing imaginal discs expressing *en-Gal4 UAS-Dcr2 UAS-RFP (red)* with either *UAS-fat-RNAi (A) or UAS-elgi-RNAi* (B-D) and *Dachs*:*GFP (D*:*GFP)* (A, B), *Vam*:*GFP* (C) or *Fat*:*GFP* (D) showing increased levels of membrane localized Dachs:GFP, Vam:GFP and Fat:GFP in the posterior compartment. A and B were imaged using the same acquisition settings. (E) Horizontal apical sections of wing imaginal discs expressing Dachs:GFP under the control of the actin5C promoter (*act>D*:*GFP)* throughout, showing increased levels of membrane localized Dachs:GFP, in homozygous *elgi*^*1*^ mutant clones, marked by absence of RFP (red). (F) Western blot showing levels of Dachs:V5 (D:V5) from third instar wing disc lysate from flies expressing *tub-Gal4 UAS-D*:*V5* without (control) or with *elgi-RNAi*. Tubulin was used as a control for loading and transfer. Scale bar is 33 μm (A,B,E), 19 μm (C) and 16.5μm (D).

To further examine the possibility that Elgi could be involved in Ds-Fat signaling, and to begin to investigate how it might influence this pathway, we examined the effect of loss of *elgi* on the key Fat signaling components, Ds, Fat, Dachs and Vam. Depletion of *elgi* by RNAi did not result in any visible effect on the localization or levels of Ds (S2 Fig). Loss of *elgi* leads to a slight increase in Fat levels (Fig 2D), but this cannot explain the increased Yki activity and wing growth observed, because over-expression of Fat decreases Yki activity and wing growth [7, 41]. In contrast, in cells lacking *elgi*, both Dachs and Vam, examined using genomic GFP-tagged transgenes, accumulate at very high levels (Fig 2B and 2C, S4A Fig). Since over-expression of Dachs or Vam can increase wing size [8, 14, 15], this could potentially account for the increased wing growth associated with loss of *elgi*.

Absence of *fat* also leads to increased Dachs levels. To compare the increases in Dachs generated by loss of *elgi* versus loss of *fat*, *elgi* or *fat* were knocked down by RNAi in the posterior compartment of wing discs expressing Dachs:GFP, which were examined using the same imaging settings for both genotypes. This revealed that apical Dachs levels appear higher in the absence of *elgi* than in the absence of *fat* (Fig 2A and 2B). However, in the absence of *fat* Dachs localizes evenly around the cell circumference at the sub-apical membrane, whereas in the absence of *elgi* Dachs localizes to the membrane in a punctate pattern, similar to its localization in wild-type cells.

To examine whether the increase in the levels of Dachs is transcriptional or post-transcriptional, we examined the influence of elgi-RNAi and *elgi*^*1*^ mutant clones on a transgene expressing Dachs:GFP under the control of the Act5c promoter. Act5c-Dachs:GFP accumulated at high levels in the apical cortex in the absence of *elgi* (Fig 2E, S4C and S4E Fig), just like Dachs:GFP expressed from its endogenous promoter, which indicates that *elgi* regulates Dachs at a post-transcriptional level. Consistent with this, western blotting of wing disc lysates revealed that loss of *elgi* leads to a significant increase in the levels of V5-tagged Dachs expressed ubiquitously under the control of a tub-Gal4 driver (Fig 2F).

### Elgi acts genetically upstream of Dachs, and in parallel to Fat

**Fig 3:**
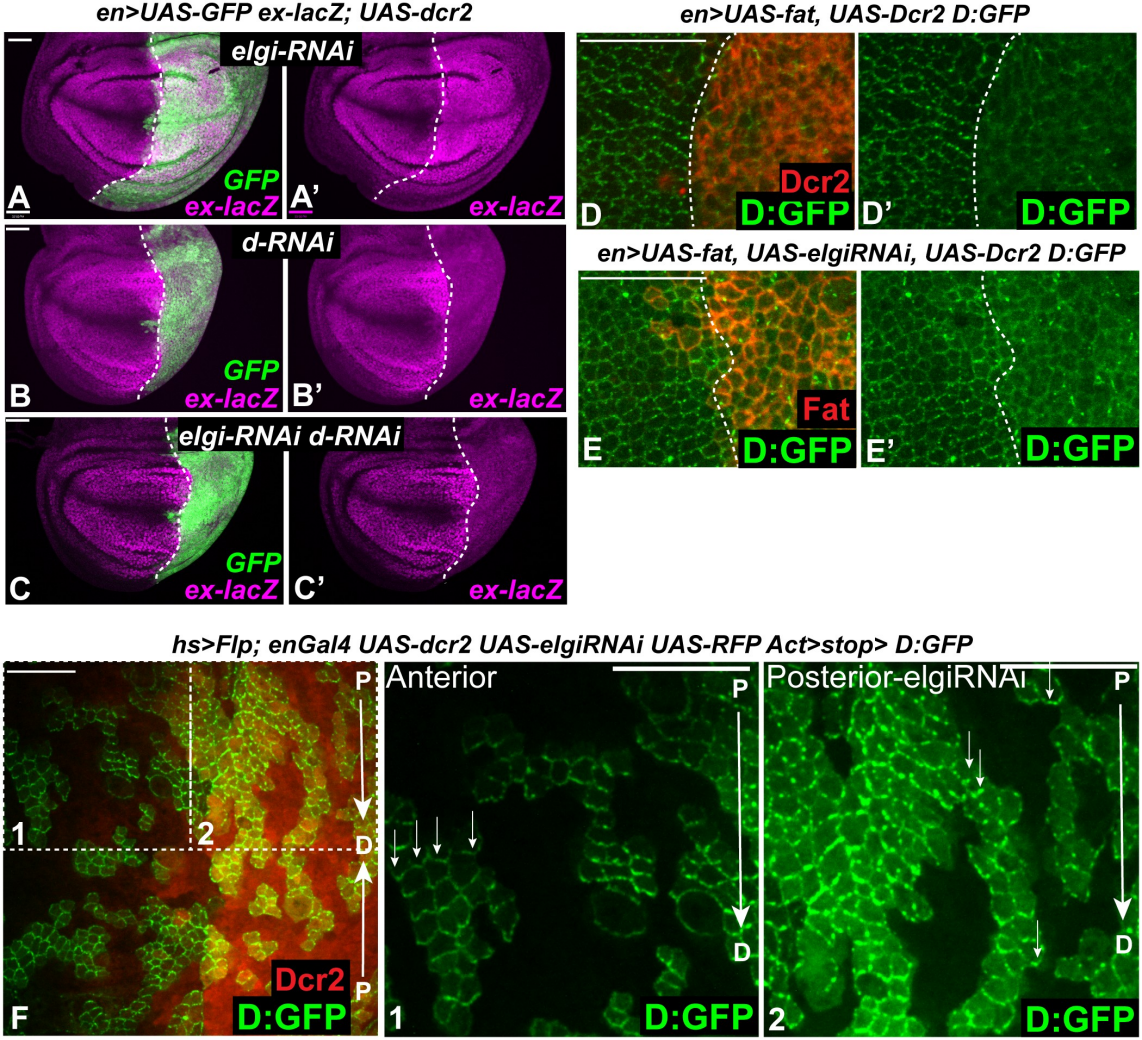
*elgi* regulates Dachs in parallel to Fat and does not affect Dachs polarity. (A-C) Third instar wing imaginal discs expressing *en-Gal4 UAS-dcr2 UAS-GFP ex-lacZ* and either *UAS-elgi-RNAi* (A, A’) or *UAS-dachs-RNAi (UAS-d-RNAi)* (B, B’) or *UAS-elgi-RNAi* and *UAS-d-RNAi* (C, C’), stained for expression of *ex-lacZ* (magenta), with posterior cells marked by expression of GFP (green). Dashed white line marks the A-P compartment boundary. Scale bar is 33 μm. (D, E) Horizontal apical sections of wing imaginal discs expressing *en-Gal4 UAS-dcr2 D*:*GFP UAS-Fat* without (D, D’) or with (E, E’) *UAS-elgi-RNAi* showing displacement of membrane localized Dachs:GFP (D:GFP) into the cytoplasm. Posterior cells are marked by expression of Dcr2 or Fat (red) Dashed white line marks the A-P compartment boundary. Scale bar is 16.5 μm. (F) Horizontal apical sections of wing imaginal discs expressing Dachs:GFP (D:GFP) in flipout clones and *elgi-RNAi* in the posterior compartment (marked by RFP, red). Arrows point in a proximal (P) to distal (D) direction. (1, 2) Magnified views of boxed areas in F with small arrows indicating preferential enrichment of D:GFP on the distal boundary of cells. Scale bar is 16.5 μm.

Mutation of upstream components of the Fat signaling pathway, including Fat, Ds, and Dco, increase Yki activity and wing growth by increasing levels of Dachs at the apical membrane [7, 8, 24]. To confirm that the increased Yki activity observed with *elgi* loss-of-function is due to increased Dachs, we investigated whether the *elgi* phenotype genetically depends upon *dachs*. Indeed, depletion of *dachs* by RNAi completely suppressed elevation of *ex-lacZ* expression by *elgi* RNAi, and wing disc cells with reduction of both proteins instead exhibit the loss of *ex-lacZ* expression characteristic of *dachs* RNAi (Fig 3A–3C).

Overexpression of Fat can displace Dachs from the membrane into the cytoplasm [8, 16]. To examine the genetic relationship between *elgi* and *fat*, we investigated the influence of *elgi* on this regulation of Dachs by Fat. Fat was overexpressed in the absence or in the presence of *elgi* RNAi in the posterior compartment of wing discs expressing Dachs:GFP. Overexpression of Fat caused a displacement of Dachs from the plasma membrane even in the presence of *elgi* RNAi (Fig 3E). Thus, Fat over-expression is epistatic to *elgi* loss of function. Together with observations that *elgi RNAi* increases Fat levels, and that loss of Elgi or loss of Fat result in different patterns of increased Dachs, this suggests that *fat* and *elgi* function in parallel to regulate Dachs.

Normally Dachs localizes in a planar polarized manner, with a preferential localization to the distal side of wing disc cells [8]. To examine whether *elgi* affects Dachs polarity, we expressed Dachs:GFP in small clones in wing discs expressing *elgi* RNAi in the posterior compartment. While loss of *elgi* leads to an increase in the levels of Dachs:GFP, it is still mostly polarized to the distal side (Fig 3F). Fat signaling regulates PCP as well as Hippo signaling, and loss of Fat results in Dachs-dependent disruption of hair polarity in the proximal wing [13, 42]. However, wings from *elgi*^*1*^ mutant flies do not exhibit any defect in hair polarity (S1 Fig). These observations are consistent with the idea that the levels of membrane localized Dachs influence Hippo signaling, whereas the polarity of membrane Dachs influences PCP, and also further support the conclusion that Elgi and Fat act in parallel to regulate Dachs.

### Elgi localizes to the cytoplasm and physically interacts with Dachs

**Fig 4:**
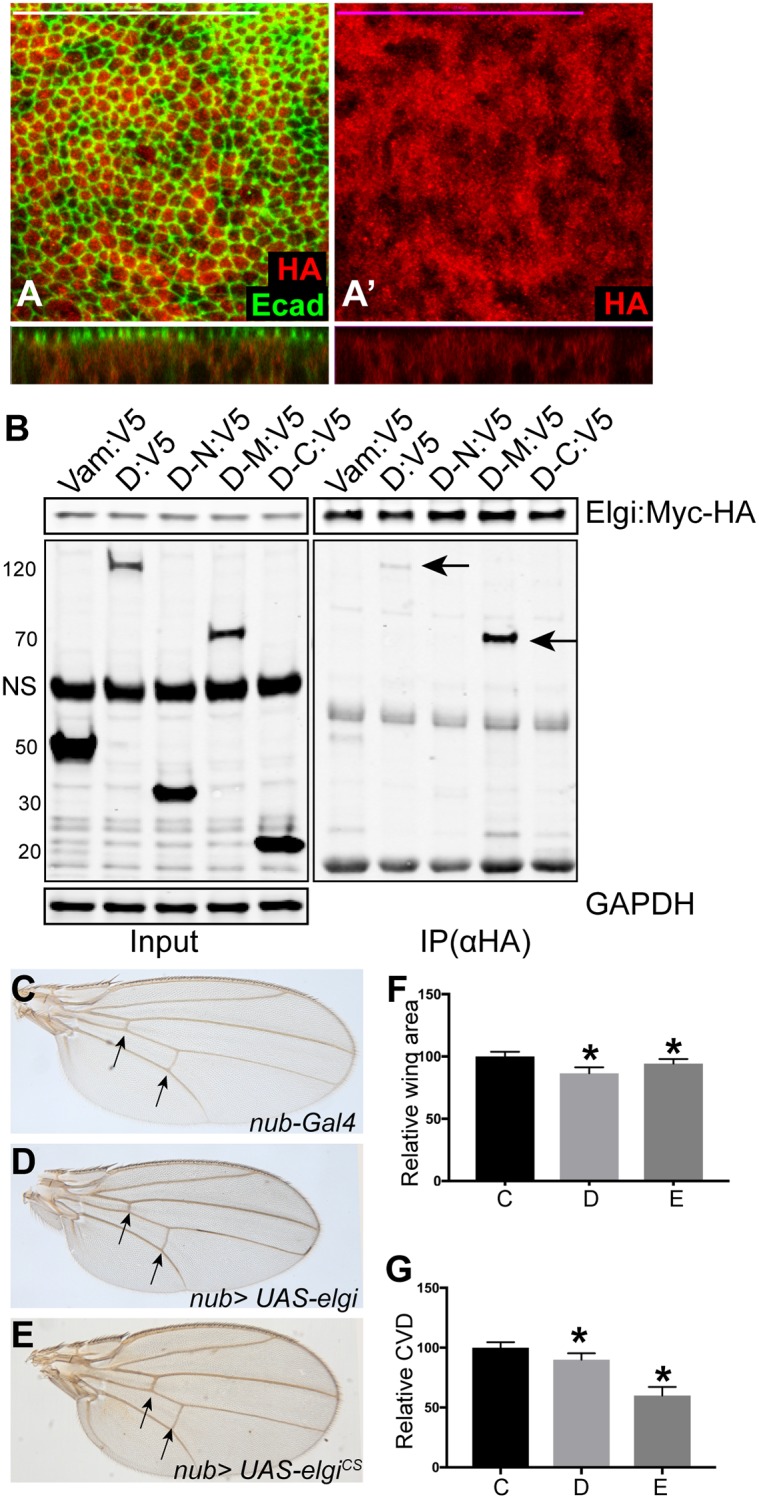
*elgi* localization and interaction with Dachs, and *elgi*^*CS*^ phenotypes. (A, A’) Horizontal apical sections (above) and vertical sections (below) of wing imaginal discs expressing *nub-Gal4 UAS-elgi-MYC-HA* and stained with anti E-cad (green) and anti HA (red) antibodies showing diffuse cytoplasmic localization of Elgi. Scale bar is 33.0 μm. (B) Western blot showing results of co-immunoprecipitation experiments on proteins co-expressed in S2 cells. V5-tagged Vam, Full length Dachs (D:V5), Dachs N terminus(D-N:V5), Dachs Middle domain (D-M:V5) or Dachs C terminus (D-C:V5) were co-expressed with full length Elgi-MYC-HA, and complexes were precipitated using Ezview red anti HA affinity resin and immunoblotted with Rabbit anti V5 and Rabbit anti HA antibodies. GAPDH was used as a control for loading and transfer in the input lanes. Arrows point to the co-immunoprecipitated bands. NS: non-specific (C-E) Adult male wings from flies expressing *nub-Gal4* (control) (C), *nub-Gal4 UAS-elgi* (D) and *nub-Gal4 UAS-elgi*^*CS*^ (E). Arrows point to the crossveins. (F-G) Histograms of relative wing areas (normalized to the average wing area of control wings) (F) and ratios of CVD to wing length, (normalized to the average CVD to wing length ratio of control wings) (G) in flies of the genotypes in panels C-E, as indicated. Data are shown as mean ± SD from measurements of 12 wings per genotype. *P<0.001, (Student’s *t* test between control and the other genotypes).

To further investigate how Elgi regulates Dachs, we examined the localization of Elgi protein. We created a GFP-tagged genomic copy of Elgi, but the extremely weak signal from this construct was insufficient to clearly establish its localization. Therefore we expressed a HA-tagged Elgi in the wing pouch under *nub-Gal4* control. Overexpression of Elgi-Myc-HA under UAS-Gal4 control results in a slightly smaller wing size (Fig 4D and 4F), opposite to the effect of *elgi* loss-of-function, while also causing a very mild decrease in cross vein spacing (Fig 4C–4G). This *UAS-elgi-Myc-HA* construct could rescue the reduced cross-vein spacing of an *elgi* mutant (S3 Fig). These observations indicate that it has Elgi activity. Anti-HA antibody staining revealed a dispersed cytoplasmic localization for Elgi (Fig 4A).

*elgi* encodes for a predicted RING domain E3 ubiquitin ligase, homologous to vertebrate RNF41 protein. Since we found that Elgi regulates the levels of Dachs and Vam, we examined whether Elgi physically interacts with them. To test this, we co-expressed Myc-HA-epitope-tagged Elgi along with V5-epitope-tagged full length Vam or Dachs, or N-terminal, middle or C-terminal fragments of Dachs, in S2 cells and conducted co-immunoprecipitation experiments. While Elgi does not interact with Vam, it can co-precipitate both full length Dachs, and the middle region of Dachs (Fig 4B). Since Dachs can influence levels of Vam [14, 15], this suggests that *elgi* could primarily affect Dachs, with the increase in Vam levels arising indirectly from the increase in Dachs levels. The middle region of Dachs is homologous to regions of myosin family proteins. Interestingly, a pulldown experiment identified Myo10A as an interactor of Elgi [43], suggesting that Elgi interacts with a subset of myosin domains.

**Fig 5:**
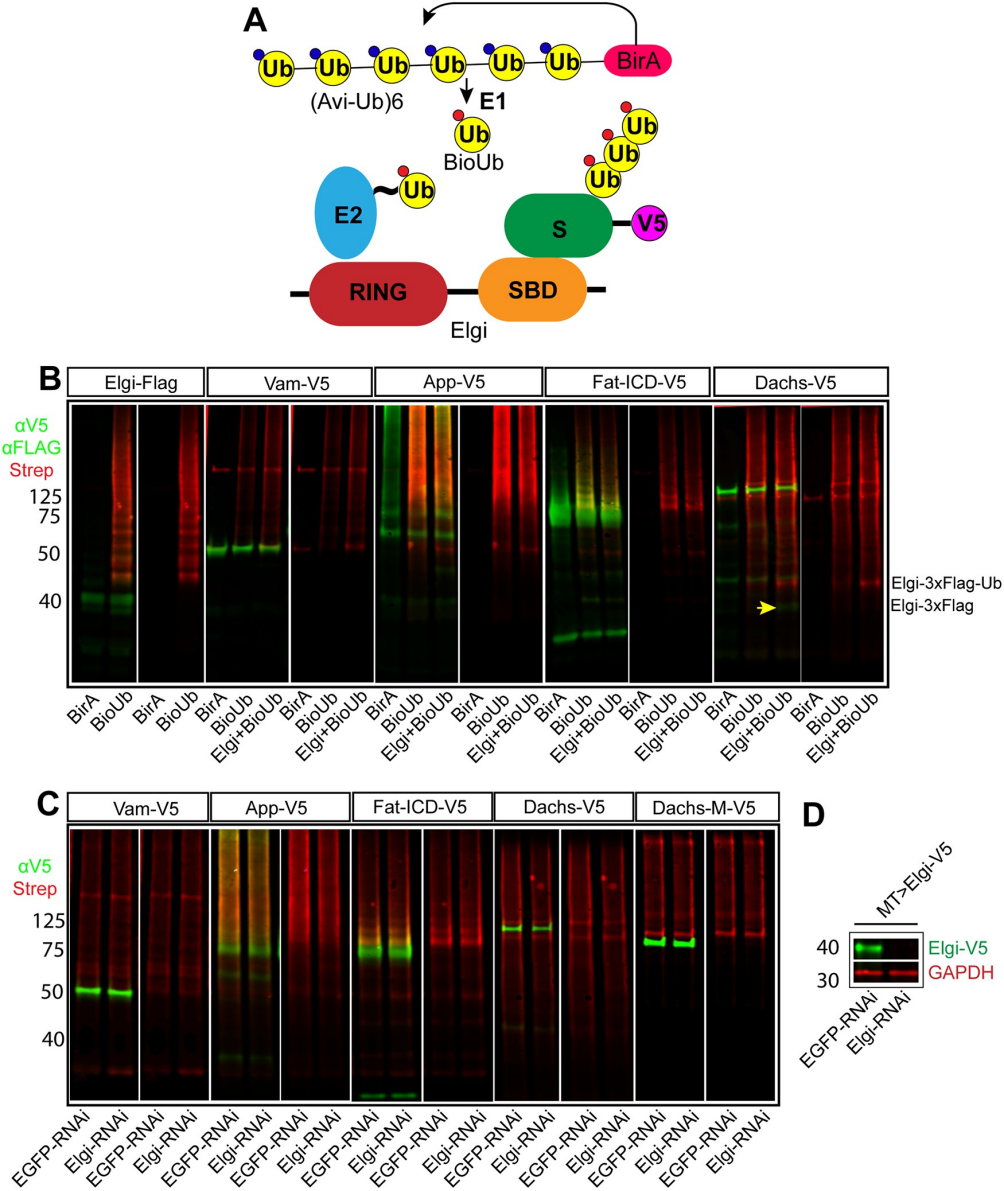
*elgi* does not ubiquitinate Vam, App, Fat-ICD or Dachs. (A) Schematic illustrating the ubiquitination assay used. BirA covalently conjugates Biotin to the fused Avi-tagged ubiquitin, which is processed by the E1 to produce biotinylated ubiquitin (BioUb), which is subsequently incorporated by the ubiquitination machinery onto the substrates. This can be detected by fluorescently conjugated streptavidin. (B) Western blot showing results of ubiquitination experiments on proteins expressed in S2 cells. For Elgi autoubiquitination, 3xFLAG tagged Elgi was co-expressed with either BirA or BirA-BioUb and immunoprecipitated using anti-Flag beads. Elgi was detected by anti-FLAG antibody. V5-tagged Full length Vam (Vam-V5), App (App-V5), Fat-ICD (Fat-ICD-V5) or Dachs (Dachs-V5) were co-expressed with either BirA alone or BirA-BioUb with or without Elgi-3xFLAG. V5-tagged proteins were immunoprecipitated from cell lysates, and detected with anti-V5 antibody. In all lanes, biotinylated Ubiquitin was detected with IRDYE680 conjugated streptavidin (Red). The yellow arrow points to a faint band of Elgi-3x-FLAG immunoprecipitated with Dachs:V5; the strong red band just above it is ubiquitinated Elgi-3x-FLAG, based on size and detection with anti-FLAG antibodies. Its appearance presumably reflects the strong association of Elgi with Dachs. (C) Western blot showing results of ubiquitination experiments on proteins expressed in S2 cells treated with dsRNA against either *EGFP* (control) or *elgi*. V5-tagged Full length Vam (Vam-V5), App (App-V5), Fat-ICD (Fat-ICD-V5), full length Dachs (Dachs-V5) or middle domain of Dachs (D-M-V5) were co-expressed with BirA-BioUb. V5-tagged proteins were immunoprecipitated from cell lysates and detected with anti-V5 antibody; Biotinylated Ubiquitin was detected with IRDYE680 conjugated streptavidin (Red). (D) Western blot showing efficiency of dsRNA-mediated depletion of *elgi* in S2 cells. V5-tagged Elgi (Elgi-V5) was expressed using MT-Gal4 with 0.5mM CuSO4 in presence of 10uM MG132 in S2 cells treated with dsRNA against either EGFP (control) or *elgi*, and whole cell lysate was examined for expression of Elgi-V5 with Rabbit anti-V5 antibody. GAPDH was used as a loading and transfer control.

**Fig 6:**
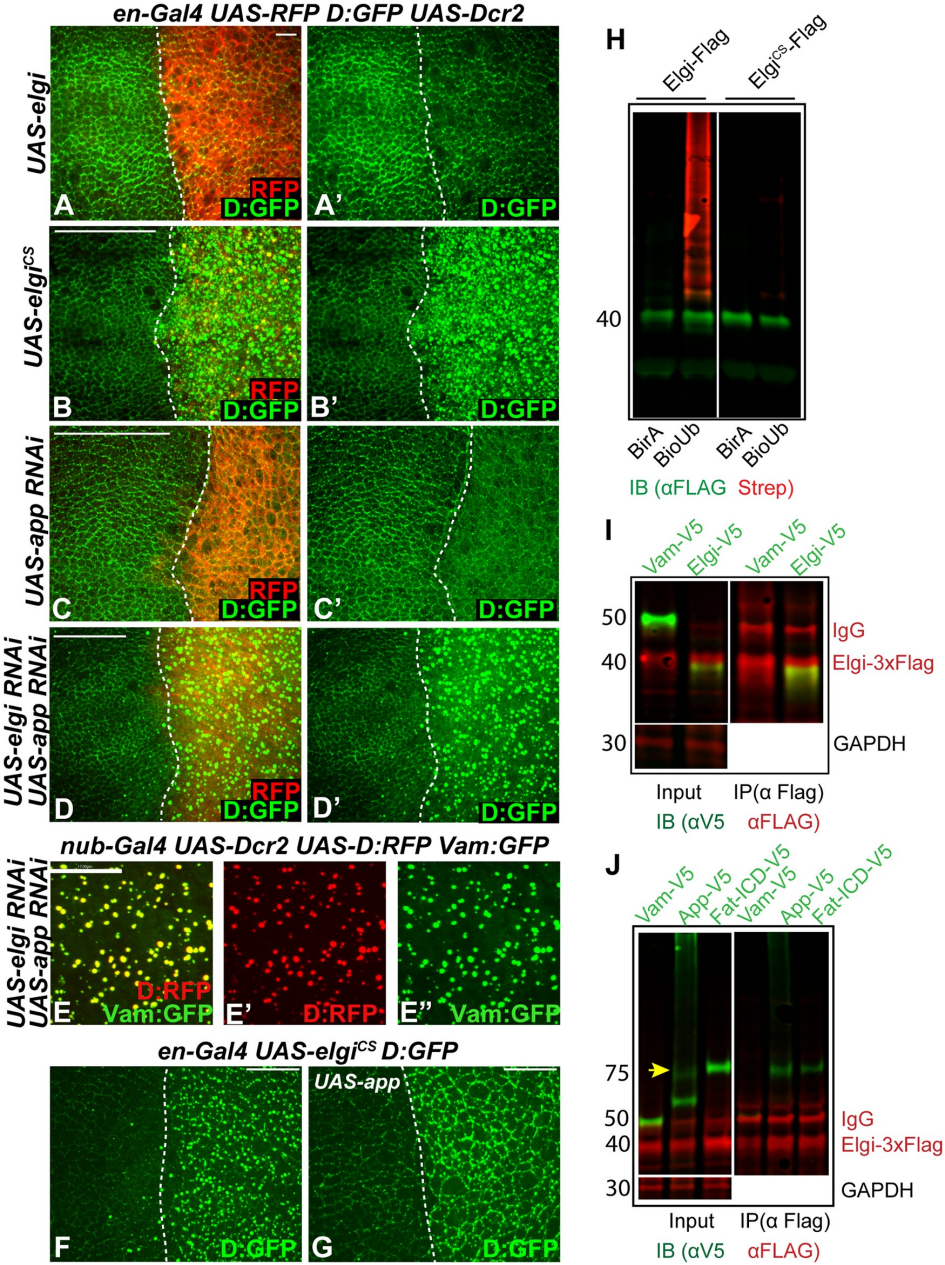
*elgi*^*CS*^ has a neomorphic activity and interferes with *app* function. (A-D) Horizontal apical sections of wing imaginal discs expressing *en-Gal4 UAS-dcr2 D*:*GFP UAS-RFP* along with *UAS-elgi-Myc-HA (UAS-elgi)* (A, A’), *UAS-elgi*^*CS*^*-Myc-HA (UAS-elgi*^*CS*^*)* (B, B’), *UAS-app-RNAi* (C, C’), or *UAS-elgi-RNAi* and *UAS-app-RNAi* (D, D’), showing the effect on Dachs:GFP (D:GFP, green) levels and localization in the posterior compartment (marked by RFP, red). Dashed white line marks the A-P compartment boundary. (E-E”) Horizontal apical sections of wing imaginal discs expressing *nub-Gal4 UAS-dcr2 UAS-D*:*RFP UAS-Vam*:*GFP UAS-elgi*-RNAi *UAS-app*-RNAi showing close up view of D:RFP (red) (E’) and Vam-GFP (green) (E’) colocalization. (F,G) Horizontal apical sections of wing imaginal discs expressing *en-Gal4 UAS-elgi*^*CS*^ *D*:*GFP* (F) or along with *UAS-app* (G) showing the effect on localization of Dachs:GFP (D:GFP) in the posterior compartment (to the right). Dashed white line marks the A-P compartment boundary. Scale bar is 6 μm in A, 33 μm in B-D, 17 μm in E and 19 μm in F and G. (H) Western blot showing results of ubiquitination experiments on proteins co-expressed in S2 cells. 3xFLAG tagged Elgi or Elgi^CS^ were co-expressed with either BirA or BirA-BioUb and immunoprecipitated using anti-Flag beads. Elgi was detected by anti-FLAG antibody and biotinylated Ubiquitin was detected with IRDYE680 conjugated streptavidin (Red). (I) Western blot showing results of co-immunoprecipitation experiments on proteins co-expressed in S2 cells. V5-tagged Vam or Elgi were co-expressed with full length Elgi-3x-FLAG, and complexes were precipitated using anti FLAG affinity resin and immunoblotted with anti-V5 and anti-FLAG antibodies. GAPDH was used as a control for loading and transfer in the input lanes. Elgi-3x-FLAG has a slower mobility compared to Elgi-V5 due to the larger epitope tag. (J) Western blot showing results of co-immunoprecipitation experiments on proteins co-expressed in S2 cells. V5-tagged Vam, App (App-V5) or Fat-ICD (Fat-ICD-V5) were co-expressed with Elgi-3x-FLAG, and complexes were precipitated using anti-FLAG affinity resin and then immunoblotted with anti-V5 and anti-FLAG antibodies. GAPDH was used as a control for loading and transfer in the input lanes. Arrow points to the full length App in the input lane.

Since Elgi physically interacts with Dachs, influences Dachs levels, and encodes a predicted ubiquitin ligase, we examined whether it can ubiquitinate Dachs. We used a recently described method that employs biotinylated-Ubiquitin, as it is highly sensitive [44]. Briefly, when avi-tagged Ubiquitin fused to *E*.*coli* biotin ligase BirA (Bio-Ub) is expressed in cells, biotinylated Ubiquitin is produced, which is then incorporated by the ubiquitination machinery (Fig 5A). We expressed Dachs:V5 in cultured *Drosophila* S2 cells along with Bio-Ub with or without Elgi, immunoprecipitated Dachs:V5 using a mouse anti-V5 antibody, and then immunoblotted with rabbit anti-V5 to detect Dachs:V5 and with fluorescently-conjugated streptavidin to detect biotinylated-Ubiquitin. As a positive control, we examined Elgi auto-ubiquitination, which was readily detected (Figs 5B and 6H). When Dachs was expressed along with Bio-Ub we detected a smear of biotinylated bands, both at higher molecular weight (presumably corresponding to poly-ubiquitinated Dachs) and at lower molecular weight (presumably corresponding to ubiquitinylated degradation products). When Elgi was co-expressed along with Dachs:V5 and Bio-Ub, this signal was not altered, aside from the detection of the co-immunoprecipitated Elgi band. These results suggest that Dachs is ubiquitinated by an E3 ligase present in S2 cells. However, because this signal does not change when Elgi is over-expressed, it appears that Elgi does not ubiquitinate Dachs. We also examined if Dachs ubiquitination in S2 cells depends upon Elgi. Using dsRNA, Elgi could be efficiently depleted in S2 cells (Fig 5D). However, *elgi* RNAi did not decrease Dachs ubiquitination (Fig 5C).

### A catalytic mutant form of Elgi has as a neomorphic activity and interferes with App function

To evaluate the potential contribution of Elgi catalytic activity to Dachs regulation, we created a mutant version of *elgi*, *elgi*^*CS*^, in which the two cysteines (C18 and C21) in the highly conserved RING domain were mutated to serines. In other E3 ubiquitin ligases, this type of mutation has been reported to abolish catalytic activity [45, 46]. Consistent with this, while wild type Elgi can auto-ubiquitinate, Elgi^CS^ lacks this activity (Fig 6H). Over-expression of *elgi*^*CS*^ results in a significant decrease in cross vein spacing in the adult wing, reminiscent of loss of *elgi* function (Fig 4E and 4G). However, unlike loss of *elgi*, it does not lead to an increase in wing size (Fig 4E and 4F). At the cellular level, expression of *elgi*^*CS*^ resulted in a dramatic increase in the levels of Dachs (Fig 6B), reminiscent of *elgi* RNAi, and opposite to the decrease in levels of Dachs observed when wild-type Elgi is over-expressed (Fig 6A). These observations suggest that Elgi^CS^ interferes with endogenous Elgi function. This could result from Elgi^CS^ binding to its substrate and preventing access to wild type Elgi. Alternatively, Elgi could function as a homodimer or heterodimer with another E3 ligase, as many RING-domain ligases are known to function as dimers [47]. To test if Elgi forms homodimers, we coexpressed 3xFlag-tagged Elgi with either V5-tagged Vam or V5-tagged Elgi in S2 cells and immunoprecipitated 3x-Flag tagged Elgi and examined for their interaction. While Elgi:3xFlag did not interact with Vam:V5 it interacted with Elgi:V5 (Fig 6I), indicating that Elgi forms homodimers, which could explain why Elgi^CS^ interferes with endogenous Elgi function.

Elgi^CS^ also causes an additional effect on Dachs, as when it is expressed, Dachs localization to the subapical membrane is diminished, and instead it accumulates in bright punctae that are dispersed throughout the cytoplasm (Fig 6B, S4G and S4H Fig). This could explain why unlike *elgi* RNAi or mutants, animals expressing *elgi*^*CS*^ do not display wing overgrowth (Fig 4E and 4F), as Dachs function depends upon its membrane localization. Expression of *elgi*^*CS*^ also does not upregulate the Yki target gene, *ex-lacZ* (S5A Fig). The formation of cytoplasmic puncta is a Dachs-specific effect, as several other proteins including Fat, Ds, Crumbs, Armadillo and Jub that normally localize to the apical cortex are not affected by *elgi*^*CS*^ (S5B–S5F Fig). Vam also gets mis-localized with Dachs when *elgi*^*CS*^ is expressed (S5G Fig, Fig 8A), but this could be because Vam forms an obligate heterodimer with Dachs [14, 15].

Since membrane localization of Dachs and Vam is promoted by App [14, 15, 33], the loss of Dachs and Vam from the apical membrane when *elgi*^*CS*^ is expressed suggested that it might interfere with App function. We also note that expression of *elgi*^*CS*^ causes rounder wings with a mild hair polarity defect in the proximal wing, similar to *app* loss-of-function (Fig 4E, S5I Fig) [33]. If Elgi^CS^ interferes with App, concomitant depletion of *elgi* and *app* should cause similar localization defects of Dachs and Vam. To examine this, we compared loss of *elgi* or *app* alone with simultaneous knockdown of both genes. Loss of *elgi* and *app* together, but not individually, leads to accumulation of Dachs and Vam in cytoplasmic punctae similar to those observed when *elgi*^*CS*^ is expressed, with Dachs and Vam co-localized (Fig 6B–6E). If *elgi*^*CS*^ interferes with *app* function, we would also predict that overexpression of App could rescue the mis-localization of Dachs induced by expression of *elgi*^*CS*^. Indeed, over-expression of App restored Dachs membrane localization in cells expressing *elgi*^*CS*^ (Fig 6G), indicating that *elgi*^*CS*^ interferes with App function. The mechanism by which Elgi^CS^ affects App is unclear. Elgi can bind to App and one of its identified substrates, the Fat-ICD (Fig 6J). However, Elgi does not detectably promote their ubiquitination (Fig 5B and 5C).

We were unable to determine the nature of the cytoplasmic accumulations of Dachs and Vam observed when *elgi* and *app* are knocked down or Elgi^CS^ is expressed. They fail to colocalize with known markers of subcellular compartments including fast recycling endosomes (Rab4), early endosomes (Rab5), late endosomes (Rab7), recycling endosomes (Rab11), lysosomes (LAMP1), multi vesicular bodies (Hrs), mitochondria (Mito-GFP) and autophagosomes (Atg8a-GFP) (S6 Fig). It is possible that they correspond to unidentified vesicular compartments. Alternatively, they could represent a transient vesicular trafficking structure that accumulates in the absence of Elgi and App. They could arise from coacervation. It is also possible that in absence of App and Elgi, Dachs accumulates in misfolded aggregates. Interestingly, palmitoylation is known to be required for the proper function of a number of chaperones [48, 49], but it remains to be examined if App affects any chaperones.

### Elgi and App primarily affect Dachs rather than Vam

**Fig 7:**
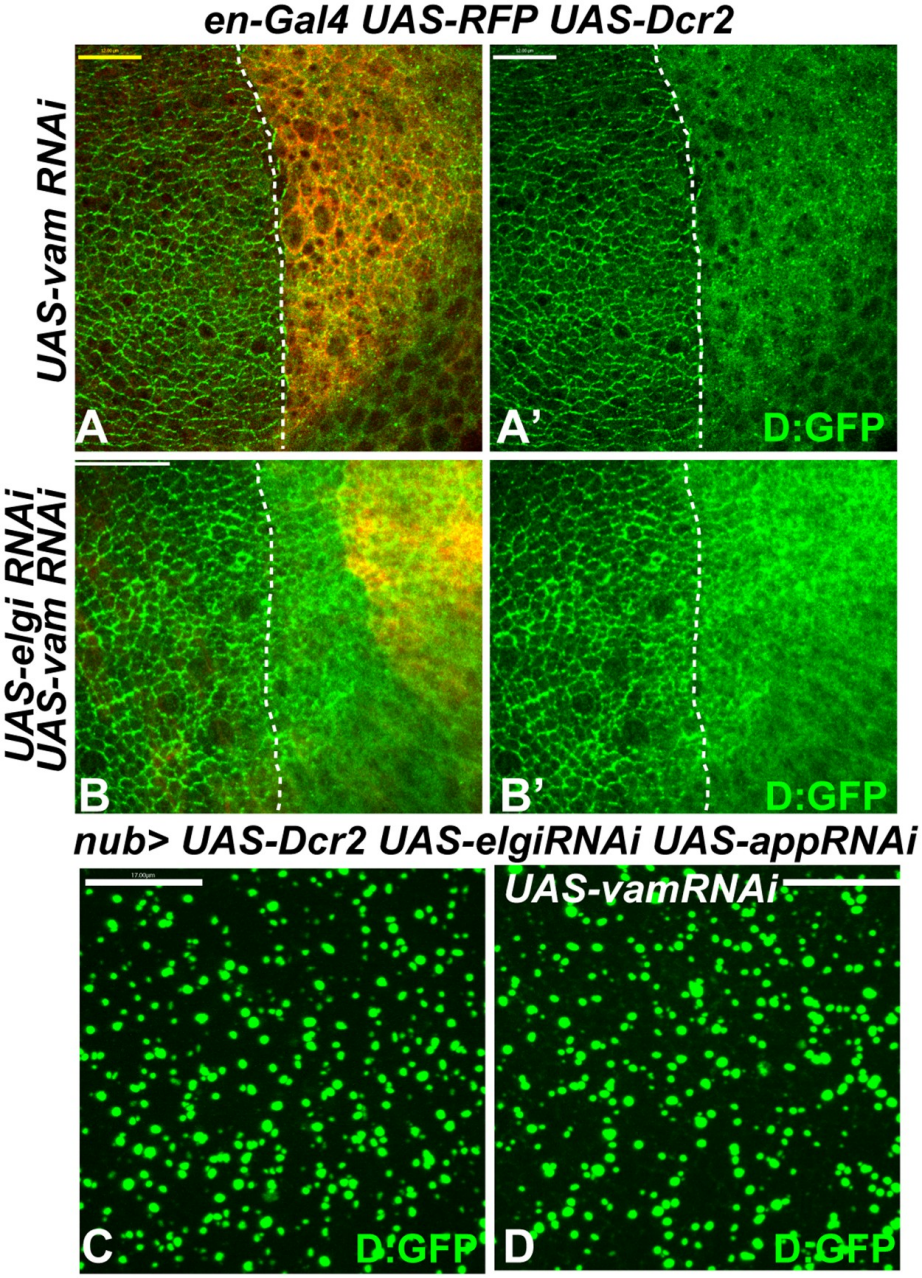
*elgi* and *app* affect Dachs in a Vam-independent manner. (A-B) Horizontal apical sections of wing imaginal discs expressing *en-Gal4 UAS-dcr2 UAS-RFP D*:*GFP* along with *UAS-vam-RNAi* (A, A’), *UAS-elgi-RNAi UAS-vam-RNAi* (B, B’), showing their effects on *D*:*GFP* localization in the posterior compartment (marked by RFP, red). Dashed white line marks the A-P compartment boundary. (C, D) Horizontal apical sections of wing imaginal discs expressing *nub-Gal4 UAS-dcr2 UAS-elgi-RNAi UAS-app-RNAi D*:*GFP* (C) or along with *UAS-vam-RNAi* (D), showing that Vam is not required for Dachs:GFP (D:GFP) mislocalization in absence of *app* and *elgi*. Scale bar is 12 μm in A, 33 μm in B and 17μm in C and D.

**Fig 8:**
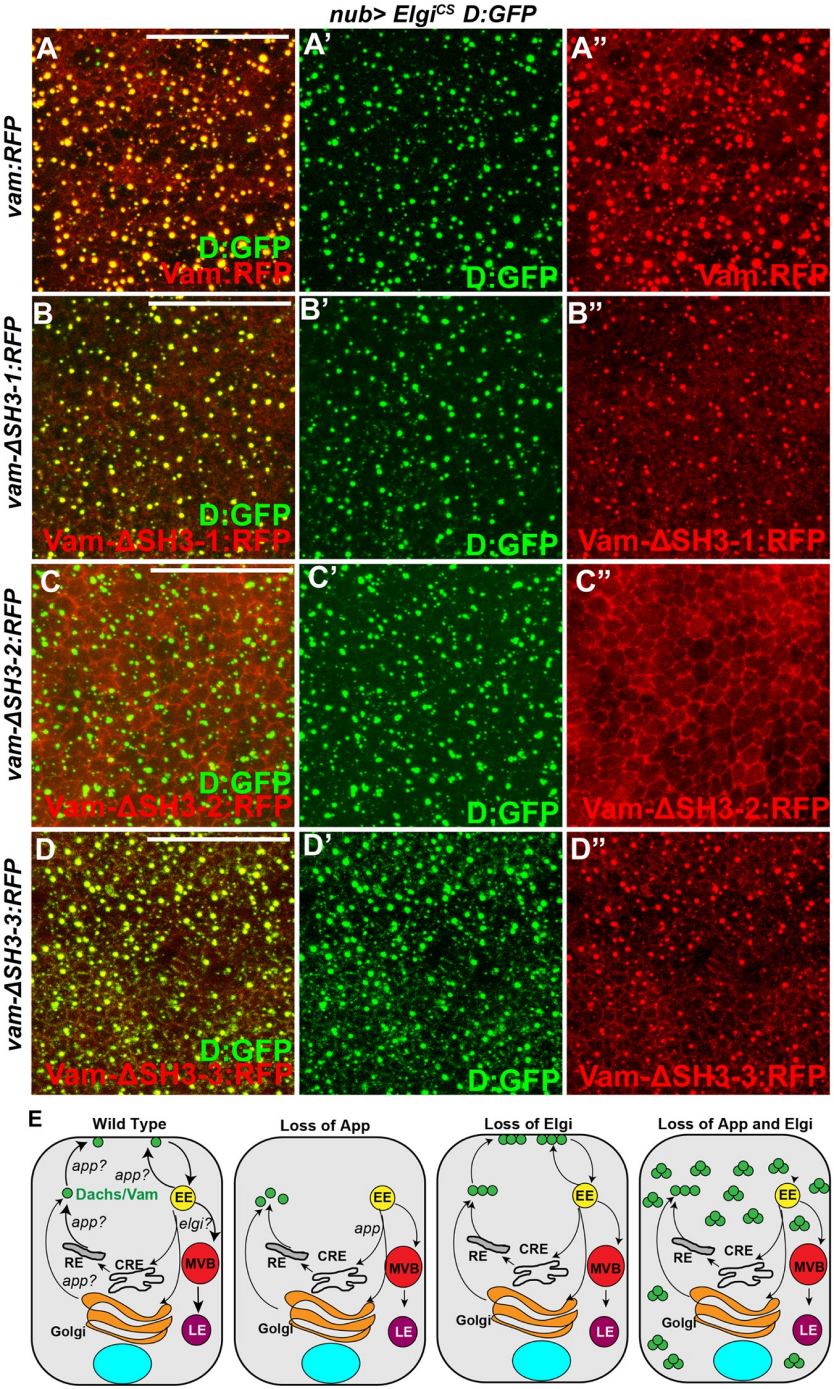
*elgi*^*CS*^ affects Dachs rather than Vam. (A-D) Horizontal apical sections of wing imaginal discs expressing *nub-Gal4 UAS-elgi*^*CS*^ *D*:*GFP* along with either *UAS-vam-RFP* (A, A', A”), or *UAS-vam-Δ-SH3-1-RFP* (B, B', B”), *UAS-vam-Δ-SH3-2-RFP* (C, C', C”) or *UAS-vam-Δ-SH3-3-RFP* (D, D', D”) showing how the different SH3 domains of Vam (red) affect its co-localization with Dachs:GFP (D:GFP) (green) in the presence of *elgi*^*CS*^. (E) Schematic illustrating model showing that under normal conditions Elgi maintains limiting levels of Dachs (green circles) which localizes to the membrane in an App-dependent manner. In absence of App, Dachs is mostly found in the cytoplasm in a diffuse manner and in absence of Elgi there is higher amount of Dachs, but the endogenous App is sufficient to promote its membrane localization. However, in absence of both, there is excessive Dachs that fails to localize to the membrane and accumulates in punctae in the cytoplasm. Elgi might regulate lysosomal trafficking of a Dachs/Vam complex, or direct proteosomal degradation of a Dachs/Vam complex. EE: early endosome, MVB: multi vesicular body, CRE: common recycling endosome, RE: recycling endosome, LE: late endosome. Scale bar is 16.5 μm in A-D.

Expression of *elgi*^*CS*^ or simultaneous depletion of *elgi* and *app* led to accumulation of both Dachs and Vam in cytoplasmic punctae (Figs 6B, 6D, 6E and 8A). App can palmitoylate Vam in S2 cells [15]. If App acts through Vam *in vivo* to regulate Dachs localization, then we would expect simultaneous depletion of *elgi* and *vam* to result in a similar mislocalization of Dachs. However, Dachs does not mislocalize to cytoplasmic punctae when *elgi* and *vam* are both knocked down (Fig 7B), suggesting that Vam may not be a bona fide substrate of App *in vivo*.

The mislocalization of both Dachs and Vam in the absence of *elgi* and *app* could be explained either by Elgi and App directly affecting both Dachs and Vam, or alternatively by only directly affecting one of them, with the other mislocalized due to the physical interaction between Dachs and Vam. To distinguish between these possibilities, we examined whether Vam is required for Dachs mislocalization in absence of *elgi* and *app*. Depletion of Vam in cells lacking *elgi* and *app* did not affect the mislocalization of Dachs (Fig 7C and 7D), indicating that Vam is not required for Dachs mislocalization. To investigate the potential requirement for Dachs in Vam mislocalization, we took advantage of the observation that the second SH3 domain of Vam is specifically required for its interaction with Dachs [14, 15]. We examined how deletion of the different SH3 domains of Vam affected its localization in presence of *elgi*^*CS*^. Full length Vam-RFP as well as Vam-RFP lacking either the first (Vam-ΔSH3-1-RFP) or third (Vam-ΔSH3-3-RFP) SH3 domains were mislocalized along with Dachs:GFP (Fig 8A, 8B and 8D), but Vam-RFP lacking the second SH3 domain (Vam-ΔSH3-2-RFP), which fails to interact with Dachs, did not mislocalize with Dachs:GFP in presence of *elgi*^*CS*^ (Fig 8C). Together, these experiments indicate that *elgi* and *app* primarily affect Dachs, and that in absence of both *elgi* and *app*, Vam gets mislocalized along with Dachs because it physically interacts with Dachs. In addition, they suggest that Vam does not seem to be the substrate of App that regulates Dachs localization.

If Vam is affected by Elgi solely through its interaction with Dachs, then Vam that cannot interact with Dachs could be more stable than wild-type Vam. To test this, we expressed either full length Vam-RFP or Vam-RFP lacking one of the SH3 domains, using a ubiquitously expressed tub-Gal4 and UAS-vam transgenes inserted at the same genomic locus. Western Blotting of wing disc lysates revealed that Vam-ΔSH3-2:RFP, which cannot interact with Dachs, is expressed at a higher level than the other Vam constructs (S7 Fig), suggesting that association with Dachs regulates Vam protein stability.

## Discussion

Our results identify the E3 ubiquitin ligase Elgi as playing a crucial role in modulating Fat-Hippo signaling. Elgi regulates the stability of Dachs and, together with App, its membrane localization. Through its effects on Dachs, Elgi also indirectly regulates the levels and localization of Vam. Our observations suggest a model (Fig 8E) where, under normal conditions, Elgi maintains limiting levels of Dachs and Vam by facilitating their degradation, and controls their localization in coordination with App. In the absence of Elgi, Dachs and Vam levels increase, but App is still sufficient for their membrane localization. However, when *elgi*^*CS*^ is expressed or when *elgi* and *app* are simultaneously depleted, the increased levels of Dachs and Vam are unable to localize to the membrane and instead accumulate in punctate structures in the cytoplasm. Due to the impact that membrane levels of Dachs have on Warts activity, Elgi influences the activity of Yki, the key transcription factor of the Hippo pathway.

Our observations indicate that Elgi regulates Dachs in parallel to Fat, and the distinct ways in which they regulate Dachs provide insight into how Dachs regulates Hippo signaling and PCP. The increase in Dachs at membranes appears greater when Elgi is knocked down than when Fat is knocked down but the increase in wing growth is greater when Fat is knocked down than when Elgi is knocked down. This might be explained by an influence of *elgi* on other proteins, which counteract the growth promoted by Dachs. Alternatively, it could be explained by the distinct patterns of Dachs membrane localization in *fat* versus *elgi* loss-of-function. In the absence of Fat, Dachs localizes to the membrane around the entire circumference of the cell, whereas in the absence of Elgi, Dachs still remains polarized. Investigations of Hippo signaling have revealed that Warts is activated in discrete complexes at the sub-apical membrane, where multiple upstream components co-localize [50, 51]. We suggest that when Dachs is membrane localized around the entire cell circumference, it is able to broadly disrupt Warts activation, whereas when Dachs is polarized, there could be regions of the sub-apical membrane where Warts activation can occur normally, resulting in a lower overall level of Yki activity than can be achieved with uniform Dachs membrane localization. The normal polarization of Dachs, together with the normal hair polarity in *elgi* mutants, is consistent with the conclusion that Fat signaling regulates PCP through the polarization of Dachs localization. Thus Elgi effectively separates the two distinct downstream branches of Fat signaling–it disrupts Hippo signaling, but leaves PCP unaffected.

Elgi is a homolog of mammalian RNF41/NRDP1, which regulates ErbB3 and ErbB4 receptors, BRUCE, BIRC6 and Parkin [52–54]. RNF41 also regulates trafficking of certain JAK2-associated type1 receptors [55]. Moreover, it can regulate PCP by ubiquitinating Dishevelled (DVL) [56]. Interestingly, RNF41 physically interacts with VANGL2 but does not ubiquitinate it [56]. Rather it ubiquitinates DVL, which is associated with VANGL2. RNF41 also stabilizes AP2S1 in an E3 ligase activity-independent manner [57].

Although Elgi can physically interact with Dachs, App and Fat-ICD, we were unable to detect direct ubiquitination of these proteins by Elgi. Thus Elgi might regulate Dachs stability through an unknown protein. Alternatively, it is possible that Elgi might function as an adapter to facilitate proteosomal or endolysosomal degradation of Dachs. For example, Elgi might directly link Dachs (or a Dachs-Vam complex) to the proteasome. Some E3 ligases, such as Stuxnet, have been reported to regulate protein degradation without ubiquitination by acting in this way [58]. If Elgi plays a role in vesicular trafficking, it might not only promote trafficking of Dachs (or a Dachs-Vam complex) to the lysosome, it could also affect the localization and levels of other proteins. The increase in Fat levels in *elgi* mutant cells is consistent with this possibility.

Dachs is also regulated by FbxL7 [16, 17], which like Elgi is predicted to be a ubiquitin ligase. It is also not clear if FbxL7 regulates Dachs by directly ubiquitinating it or instead by affecting vesicular trafficking. However, FbxL7 differs from Elgi in that FbxL7 regulates both the levels and the polarity of Dachs, and in that FbxL7 itself exhibits polarized membrane localization. The presence of multiple mechanisms to limit amounts of Dachs emphasize the importance of strict control over Dachs levels.

The palmitoyltransferase App is critical for membrane localization of Dachs [33]. Although App can palmitoylate Vam and the Fat ICD when overexpressed [15, 34], the mechanism by which it regulates Dachs localization in vivo is still not clear. Our studies revealed that concomitant loss of Elgi and App leads to accumulation of Dachs and Vam in cytoplasmic punctae. The detection of these puncta might reflect a role for App in trafficking of Dachs and Vam. In the presence of wild-type Elgi, the low levels of Dachs could preclude detection of this trafficking defect, but the dramatic increase in Dachs levels in the absence of *elgi* could exacerbate accumulation of Dachs/Vam in vesicles and make them easier to detect. Consistent with this possibility, basal puncta of Dachs were detected when Elgi was depleted and Dachs was expressed using the *act5C* promoter. Alternatively, Elgi and App might impair distinct activities that are redundantly required for trafficking of Dachs and Vam. Palmitoylation is well known to play a key role in vesicular trafficking [59]. Further characterization of the nature of these cytoplasmic accumulations of Dachs could help understand the exact mechanism by which App regulates Dachs localization.

Our genetic experiments also suggest that Vam may not be a relevant substrate of App in vivo, as concomitant loss of *vam* and *elgi* does not result in the accumulations of Dachs observed in the absence of *elgi* and *app*. This is consistent with the finding that mutating a predicted palmitoylation site within Vam does not abrogate its membrane localization [14]. Our experiments also revealed that *elgi* and *app* primarily affect Dachs, and only indirectly affect Vam through its physical association with Dachs. This is consistent with the observation that Elgi associates with Dachs but not with Vam. Interestingly, Vam lacking the second SH3 domain is expressed at a higher level compared to the full length Vam or Vam lacking the first or third SH3 domains. This suggests that Elgi-mediated degradation normally maintains Vam at low levels by acting on the Dachs-Vam heterodimer.

## Materials and methods

### *Drosophila* strains

The following previously described alleles and transgenes were used, *elgi*^*1*^, *elgi*^*2*^ [40], *Df(3L)BSC575* (BL27587), *en-Gal4* (BL30564), *nub-Gal4 (BL25754)*, *tub-Gal4*, *UAS-dcr2*, *ex-LacZ* (BL44248), *ban-lacZ* (BL10154), *UAS-mCD8*:*RFP (gift of G*. *Morata*, *Universidad Autónoma de Madrid*, *Madrid)*, *Dachs*:*GFP* [31], *Ds*:*GFP* [21], *UAS-Dachs*:*V5* [8], *UAS-Fat* [41], *UAS-vam-RNAi* (BL38263), *UAS-fat-RNAi (VDRC 9396)*, *UAS-d-RNAi (VDRC 12555)*, *UAS-app*, *UAS-app-RNAi* [33], *Vam*:*GFP*, *UAS-vam-3x-FLAG-RFP*, *UAS-vam-ΔSH3-1-3x-FLAG-RFP*, *UAS-vam-ΔSH3-2-3x-FLAG-RFP*, *UAS-vam-ΔSH3-3-3x-FLAG-RFP* and *ACT>Stop>Vam-3x-FLAG-GFP* [14], *Jub*:*GFP* [60], *UAS-D*:*RFP*, *UAS-elgi RNAi* (VDRC109617, NIG 17033R-3) *UAS-Mito-GFP* (BL8442), *UAS-atg8a-GFP* (52005), *LAMP1-YFP (DGGR 115517)*, *Rab4-YFP (BL62542)*, *Rab5-YFP (BL62543)*, *Rab7-YFP (BL62545)*, *Rab11-YFP (BL62549)*. The transgenes *UAS-elgi-Myc-HA and UAS-elgi*^*CS*^*-Myc-HA* were created in this study.

### Immunohistochemistry

Dissected wing discs were fixed in 4% paraformaldehyde for 10 minutes at room temperature and stained with primary antibodies, rat anti-E-cad (1:400, DSHB DCAD2), rat-anti-Fat (1:4000) (Feng and Irvine, 2009), rat-anti-Ds (1: 5000) (Ma et al., 2003), mouse-anti-Crb (1:200, DSHB), mouse-anti-Arm (1:200, DSHB) mouse anti-β-gal (1:400, DSHB JIE7-c), Guinea pig anti-Hrs (gift from Hugo Bellen)(1:200); and secondary antibodies, donkey anti-rat-647 (1:100, Jackson, 712-605-150) and donkey anti-rat-Cy3 (3:400, Jackson, 712-165-150). GFP and RFP were detected by autofluorescence. Confocal images were captured using a Leica SP8 confocal microscope.

### Molecular biology

To create pUAST-elgi-Myc-HA, *elgi* cDNA was PCR amplified with elgi-forward 5’TGAATAGGGAATTGGGAATTCATGGGCTACGATGTGAATCGCTT3’ and elgi-MYC-HA-rev CGCAAGATCTGTTAACGAATTCCTAAGCGTAATCTGGAACATCGTATGGGTACAGATCCTCTTCTGAGATGAGTTTTTGTTCCTCGATGCCATGGGCGAAG3’ primers and cloned into pUAST plasmid digested with EcoRI by Gibson assembly. To create pUAST-elgi^CS^-Myc-HA, *elgi* cDNA was PCR amplified with elgi^CS^ forward primer, 5’TGAATAGGGAATTGGGAATTCATGGGCTACGATGTGAATCGCTTTCAGGGGGAGGTGGACGAGGAGCTCACCTCTCCCATCTCCTCCGGAGTGCTT 5’ and elgi-MYC-HA-rev primer and cloned into pUAST plasmid digested with EcoRI by Gibson assembly. All plasmids were sequence verified. pUAST-elgi-V5 and pUAST-elgi-3xFLAG were similarly created using the elgi-forward primer and elgi-V5 reverse 5’GCAAGATCTGTTAACGAATTCCTAGGTGCTGTCCAGGCCCAGCAGGGGGTTGGGGATGGGCTTGCCCTCGATGCCATGGGCGAAG 3’ and elgi-FLAG-reverse 5’ GCAAGATCTGTTAACAATTCTTACTTGTCATCGTCATCCTTGTAATCGATGTCATGATCTTTATAATCACCGTCATGGTCTTTGTAGTCCTCGATGCCATGGGCGAAG primers respectively. pACU2-app:V5 was created by amplifying the app CDNA using forward 5’gttcaattacagctcgaattcATGAATCTGCTGTGCTGCTGTTG 3’ and reverse primer 5’ cgcagatctgttaacgaattcTTAGGTGCTGTCCAGGCCCAGCAGGGGGTTGGGGATGGGCTTGCCGACTATGGCCACGTTTGTGGTC and and cloned into pACU2 plasmid digested with EcoRI, by Gibson assembly. To create pUAST-Vam-V5, Vam cDNA was amplified with Vam-for 5’TGAATAGGGAATTGGGAATTCATGGCATTTCTTTGCCCCGT3’ and Vam-V5-Rev 5’GCAAGATCTGTTAACGAATTCTTACGTAGAATCGAGACCGAGGAGAGGGTTAGGGATAGGCTTACCAAGGCTGGTCATCGCGGGTGGT3’primers and cloned into pUAST plasmid digested with EcoRI, by Gibson assembly. To create pUAST-D-RFP, Dachs cDNA was amplified with D-for 5’TGAATAGGGAATTGGGAATTCATGTTGACTACGACGATCTGGACAG 3’ and D-Rev 5’ GCTCTTCGCCCTTAGACACCATTTTACTGAGCGTCATGAACTGGAAGG 3’ primers. Tag-RFP was amplified with RFP-For 5’ ATGGTGTCTAAGGGCGAAGAG and RFP-Rev 5’ TCCTTCACAAAGATCCTCTAGATCAATTAAGTTTGTGCCCCAGTTTGC3 and cloned into pUAST plasmid digested with EcoRI and XbaI by Gibson assembly. All plasmids were verified by sequencing.

### dsRNA production

Small fragments of elgi or EGFP coding sequences were selected using SnapDragon tool to avoid any off-target effect. For generating the template for dsRNA synthesis for elgi, elgi T7 forward 5’TAATACGACTCACTATAGGGGGATGCATTAACGAGTGGCTAACC 3’ and T7 Reverse 5’ TAATACGACTCACTATAGGGATAGTCAGCTGCTGGTCGGTG 3’ primers were used. For dsRNA synthesis for EGFP, a fragment was amplified with the EGFP-T7 forward 5’TAATACGACTCACTATAGGGACGTAAACGGCCACAAGTTC 3’ and EGFP-T7 reverse 5’ TAATACGACTCACTATAGGGTGTTCTGCTGGTAGTGGTCG 3’ primers. dsRNA synthesis was carried out using MEGAscript T7 transcription Kit (Invitrogen), following manufacturer’s instruction. After synthesis the dsRNA was purified using RNAeasy mini Kit (Quiagen).

### Cell culture, immunoprecipitation and western blotting

S2 cells were grown in Schneider’s media and transfected with plasmids aw-GAL4, pUAST-D-:V5 [8] pUAST-D-N:V5, pUAST-D-M:V5, pUAST-D-C:V5 [10], pUAST-Vam-V5 [14] along with pUAST-elgi-Myc-HA, using effectene transfection reagent (Quiagen) following the manufacturer’s instruction. Cells were lysed in RIPA buffer supplemented with protease inhibitor cocktail and phosphatase inhibitor cocktail (Calbiochem). Cell lysates were incubated with affinity resin conjugated with anti HA antibody (Sigma) for two hours at 4 °C, following which they were washed four times. Proteins samples were boiled with Laemlii buffer and subjected to SDS-PAGE using 4–15% gradient gels (Bio Rad). Western transfer was carried out using Trans-Blot Turbo transfer system (Bio Rad) and immunoblotting was performed using the primary antibodies, rabbit-anti-V5 (Bethyl Laboratories, 1:5000), mouse anti-V5 (Invitrogen, 1:5000), mouse-anti-GAPDH (Covance, 1:1000), mouse anti α-Tubulin (1:20,000), mouse anti FLAG (Sigma, 1:5000) Rabbit anti FLAG (Sigma, 1:5000) and Guinea Pig anti App (gift from Seth Blair, 1:5000); and as secondary antibodies goat anti-mouse 680 (Li-Cor, 926–68,020), goat anti-rabbit-680 (Li-Cor, 926–68,021) and Donkey anti-Gunea Pig-780 (Li-Cor, 926–32411), all at a dilution of 1:10,000. Blots were scanned using an Odyssey Imaging System (Li-Cor). Co-immunoprecipitation experiments in Fig 6I and 6J were performed following similar protocols where S2 cells were transfected with aw-Gal4, pUAST-Elgi-3x-FLAG together with either pUAST-Vam-V5 or pUAST-Elgi-V5 (Fig 6I) or pACU2-App-V5 or pUAST-Fat-ICD-V5 (Fig 6J). Elgi-3x-Flag was immunoprecipitated using EZ view Red FLAG M2 affinity Gel and immunoblotted with Mouse anti-FLAG and Rabbit-anti-V5 primary antibodies and donkey anti-mouse 680 and donkey anti-rabbit 780 secondary antibodies.

### Ubiquitination assay

Ubiquitination assays were carried out as previously described [44]. Briefly, S2 cells were transfected with either pUAST-Vam:V5, pACU2-App:V5, pUAST-Fat-ICD:V5 or pUAST-D:V5 with or without pUAST-BioUb-BirA and pUAST-elgi-3x-FLAG along with pmt-Gal4. After incubation for 72 hours, protein expression was induced by adding 0.5mM CuSO_4_. In all ubiquitination experiments, to prevent degradation of the ubiquitinated products, cells were treated with the proteasome inhibitor MG132 (10uM) at the same time as CuSO_4_ was added. After overnight incubation, cells were lysed in RIPA buffer supplemented with protease inhibitor cocktail (Roche) and phosphatase inhibitor cocktail (Calbiochem) and 10mM N-ethyl Maleimide (deubiquitinase inhibitor). Cell lysates were incubated with affinity resin conjugated with mouse anti-V5 antibody (Sigma) for two hours at 4 °C, following which they were washed four times. After washing, samples were boiled in Laemmli buffer and subjected to SDS-PAGE followed by Western transfer. Immunoblotting was performed using rabbit anti-FLAG and rabbit anti-V5 primary antibodies and goat anti-rabbit-800 (Licor) secondary antibody. IRDye-680RD-Streptavidin (Licor 925–68079) was used at 1:10000 dilution to detect biotin, and the blots were scanned using an Odyssey imaging system (LiCor). Elgi autoubiquitination was performed following a similar protocol, where S2 cells were transfected with pUAST-Elgi-3x-Flag or pUAST-Elgi^CS^-3x-Flag along with pUAST-BirA or pUAST-BioUb-BirA and Elgi-3x-Flag or Elgi^CS^-3x-Flag was immunoprecipitated using EZ view Red FLAG M2 affinity Gel (Sigma) and immunoblotted with Rabbit-anti-FLAG (Sigma) and above described secondary antibody and streptavidin.

For RNAi, cells were resuspended in serum free Schneider’s medium at a concentration of 1-4x10^5^ cells/ml and to 1ml of of cell suspension, 30μg of either EGFP-shRNA or elgi-shRNA was added and incubated for 30 minutes, following which 1ml of fresh medium containing 20% serum was added. Immediately, they were transfected with either pUAST-Vam:V5, pACU2-App:V5, pUAST-Dachs:V5, or pUAST-Fat-ICD:V5 plasmid along with the pMT-Gal4 and pUAST-BirA-bioUB plasmids, using Effectene transfection reagent (Quiagen) following manufacturer’s protocol. After incubation for 72 hours, protein expression was induced by adding 0.5mM CuSO_4_, in the presence of 10uM MG132. After overnight inductions the cells were collected and the proteins of interest were immunoprecipitated and processed as described above.

#### Statistical analyses

Statistical significance was calculated using Graphpad Prism Software. For pairwise comparisons we used t test.

## Supporting information

S1 Fig *elgi* does not affect wing hair polarity. Close up of proximal wing areas showing the normal orientation of hairs from control (A), homozygous *elgi*^*1*^ (B), and *elgi*^*1*^*/Df(3L)BSC575* (C) flies.
(TIF)

S2 Fig *elgi* does not affect Ds levels or localization. Horizontal apical sections of wing imaginal discs expressing *en-Gal4 UAS-dcr2 UAS-GFP (green) UAS-elgi-RNAi* and stained with anti-Ds antibody (red) showing no effect on the levels or localization of Ds in the posterior compartment (marked by GFP, green). Dashed white line marks the A-P compartment boundary. Scale bar is 33.00μm.
(TIF)

S3 Fig UAS-*elgi* can rescue the cross-vein spacing pehenotype of *elgi*^*1*^ mutants. (A-D) Adult male wings from UAS-*elgi-Myc-HA/+; elgi*^*1*^*/+* (control) (A), UAS-*elgi-Myc-HA/+; elgi*^*1*^ (B), *nub-Gal4* UAS-*elgi-Myc-HA/+; elgi*^*1*^*/TM6b* (C) and *nub-Gal4* UAS-*elgi-Myc-HA/+; elgi*^*1*^*/TM6b* (D) flies. Arrows point to the crossveins. (E) Histogram of the ratio of crossvein distance (CVD) to wing span, (normalized to the average CVD/wing span in control wings in flies of the genotypes in panels A-D, as indicated. Error bars indicate Standard deviation. **** (p<0.0001), *NS* not significant.
(TIF)

S4 Fig Comparison of the effects of *elgi*^*1*^ mutation, *elgi*-RNAi and elgi^CS^ on genomic and *Act5C*-driven Dachs:GFP. (A-D) Horizontal apical (A, A’, C, C’), basal (B, B’, D, D’), and vertical (A1, A2, C1, C2) sections of wing imaginal discs expressing *en-Gal4 UAS-dcr2 UAS-RFP (red) UAS-elgi-RNAi*, with either *Dachs*:*GFP (D*:*GFP)* (A, B) or *act5C* promoter driven *Dachs*:*GFP (act>D*:*GFP)* (C, D) showing increased levels of membrane localized Dachs:GFP (green), in the posterior compartment (marked by red). In D and D’ some punctate cytoplasmic accumulations of Dachs:GFP is seen in the basal sections. (E,F) Horizontal apical (E,E’), basal (F, F’) and vertical (E1, E2) sections of wing imaginal discs expressing Dachs:GFP under the control of the *actin5C* promoter (*act>D*:*GFP)* throughout, showing increased levels of membrane localized Dachs:GFP (E, E’) as well as punctate cytoplasmic accumulations of Dachs:GFP in the basal sections (F, F’) in homozygous *elgi*^*1*^ mutant clones, marked by absence of RFP (red). E-cad is shown in blue. (G-H) Horizontal apical (G,G’), basal (H,H’) and vertical (G1,G2) sections of wing imaginal discs expressing *en-Gal4 UAS-dcr2 D*:*GFP UAS-RFP* along with *UAS-elgi*^*CS*^, showing the effect on Dachs:GFP (D:GFP)(green) levels and localization in the posterior compartment (marked by RFP, red). E-cad is shown in blue. Dashed white line marks the A-P compartment boundary. Scale bar is 16.5 μm in all panels.
(TIF)

S5 Fig *elgi*^*CS*^ does not activate Yki or affect localization of several other proteins. (A, A’) Third instar wing imaginal discs expressing *en-Gal4 UAS-GFP ex-lacZ UAS-elgi*^*CS*^ stained for expression of *ex-lacZ* (magenta), with posterior cells marked by expression of GFP (green). Dashed white line marks the A-P compartment boundary. (B-F) Horizontal apical sections of wing imaginal discs expressing *en-Gal4 UAS-dcr2 UAS-elgi*^*CS*^ along with *Fat*:*GFP* (B, B’), *Ds*:*GFP* (C, C’), *Jub*:*GFP* (D, D’) or stained for Crumbs (E, E’) or armadillo (Arm) (F, F’) showing that it does not affect their localization in the posterior compartment (marked by Dcr2 staining, red). Dashed white line marks the A-P compartment boundary. (G-G”) Horizontal sections of wing imaginal discs expressing *nub-Gal4 UAS-elgi*^*CS*^ along with *D*:*GFP* and *Vam*:*RFP* showing that Vam:RFP (red) (G”) gets mislocalized with Dachs:GFP (D:GFP, green) (G’) in presence of Elgi^CS^. Scale bar is 33μm in A-F and 16.5 μm in G. (H, I) Close up of proximal wing areas showing the orientation of hairs from flies carrying *nub-Gal4* alone (H) or in combination with *UAS-elgi*^*CS*^ (I). (J) Western blot showing levels of App from third instar wing disc lysate from flies expressing *tub-Gal4* alone or with *UAS-*app or UAS-*elgi-RNAi*. Lysates from flies expressing *tub-Gal4 UAS-*app serves as positive control. Tubulin was used as a control for loading and transfer.
(TIF)

S6 Fig Dachs punctae formed in absence of *elgi* and *app* do not co-localize with known compartment markers. (A-E) Horizontal sections of wing imaginal discs expressing *nub-Gal4 UAS-dcr2 UAS-elgi-RNAi UAS-app-RNAi UAS-D*:*RFP* along with Rab4:YFP (green) (A, A’,A"), Rab5:YFP (green) (B, B’,B") Rab7:YFP (green) (C, C’,C"), Rab11:YFP (green) (D, D’,D") or LAMP1-YFP (green) (E,E’,E") showing that the cytoplasmic accumulations of Dachs:RFP (D:RFP, red) in absence of *elgi* and *app* do not colocalize with these markers. (F,G) Horizontal sections of wing imaginal discs expressing *en-Gal4 UAS-elgi*^*CS*^ *UAS-D*:*RFP* (red) along with *UAS-Mito-GFP* (green) (F,F’,F") or *UAS-atg8a-GFP* (green) (G, G’,G"). (H, H’, H”) Horizontal sections of wing imaginal discs expressing *en-Gal4 UAS-elgi*^*CS*^, *Dachs*:*GFP* (green) stained with anti-Hrs antibody (red). Scale bar is 33 μm in all panels.
(TIF)

S7 Fig Effect of different SH3 domains on relative expression levels of Vam. Western blot showing expression levels of full length Vam:RFP or Vam:RFP lacking the individual SH3 domains, from third instar wing disc lysate from flies expressing *tub-Gal4* along with *UAS-Vam*:*RFP*, *UAS-Vam-Δ-SH3-1-RFP*, *UAS-Vam-Δ-SH3-2-RFP* or *UAS-Vam-Δ-SH3-3-RFP*, with all the Vam transgenes inserted at the same genomic location and detected by αRFP antibody. Tubulin was used as a control for loading and transfer.
(TIF)

## References

[1] Staley BK, Irvine KD. Hippo signaling in Drosophila: recent advances and insights. Developmental dynamics: an official publication of the American Association of Anatomists. 2012;241(1):3–15. 10.1002/dvdy.22723 . 22174083 PMC3426292

[2] Irvine KD, Harvey KF. Control of organ growth by patterning and hippo signaling in Drosophila. Cold Spring Harbor perspectives in biology. 2015;7(6). 10.1101/cshperspect.a019224 . 26032720 PMC4448604

[3] Matis M, Axelrod JD. Regulation of PCP by the Fat signaling pathway. Genes & development. 2013;27(20):2207–20. 10.1101/gad.228098.113 . 24142873 PMC3814641

[4] Sharma P, McNeill H. Regulation of long-range planar cell polarity by Fat-Dachsous signaling. Development. 2013;140(18):3869–81. 10.1242/dev.094730 . 23946440

[5] Thomas C, Strutt D. The roles of the cadherins Fat and Dachsous in planar polarity specification in Drosophila. Developmental dynamics: an official publication of the American Association of Anatomists. 2012;241(1):27–39. 10.1002/dvdy.22736 . 21919123

[6] Blair S, McNeill H. Big roles for Fat cadherins. Current opinion in cell biology. 2018;51:73–80. Epub 2017/12/20. 10.1016/j.ceb.2017.11.006 . 29258012 PMC5949260

[7] Cho E, Feng Y, Rauskolb C, Maitra S, Fehon R, Irvine KD. Delineation of a Fat tumor suppressor pathway. Nature genetics. 2006;38(10):1142–50. 10.1038/ng1887 . 16980976

[8] Mao Y, Rauskolb C, Cho E, Hu WL, Hayter H, Minihan G, et al Dachs: an unconventional myosin that functions downstream of Fat to regulate growth, affinity and gene expression in Drosophila. Development. 2006;133(13):2539–51. 10.1242/dev.02427 . 16735478

[9] Mao Y, Tournier AL, Bates PA, Gale JE, Tapon N, Thompson BJ. Planar polarization of the atypical myosin Dachs orients cell divisions in Drosophila. Genes & development. 2011;25(2):131–6. 10.1101/gad.610511 . 21245166 PMC3022259

[10] Rauskolb C, Pan G, Reddy BV, Oh H, Irvine KD. Zyxin links fat signaling to the hippo pathway. PLoS biology. 2011;9(6):e1000624 10.1371/journal.pbio.1000624 . 21666802 PMC3110180

[11] Vrabioiu AM, Struhl G. Fat/Dachsous Signaling Promotes Drosophila Wing Growth by Regulating the Conformational State of the NDR Kinase Warts. Developmental cell. 2015;35(6):737–49. Epub 2015/12/26. 10.1016/j.devcel.2015.11.027 . 26702832 PMC4709125

[12] Ayukawa T, Akiyama M, Mummery-Widmer JL, Stoeger T, Sasaki J, Knoblich JA, et al Dachsous-dependent asymmetric localization of spiny-legs determines planar cell polarity orientation in Drosophila. Cell reports. 2014;8(2):610–21. 10.1016/j.celrep.2014.06.009 . 24998533

[13] Ambegaonkar AA, Irvine KD. Coordination of planar cell polarity pathways through Spiny-legs. eLife. 2015;4 Epub 2015/10/28. 10.7554/eLife.09946 . 26505959 PMC4764577

[14] Misra JR, Irvine KD. Vamana Couples Fat Signaling to the Hippo Pathway. Developmental cell. 2016;39(2):254–66. Epub 2016/10/26. 10.1016/j.devcel.2016.09.017 . 27746048 PMC5102026

[15] Zhang Y, Wang X, Matakatsu H, Fehon R, Blair SS. The novel SH3 domain protein Dlish/CG10933 mediates fat signaling in Drosophila by binding and regulating Dachs. eLife. 2016;5 Epub 2016/10/04. 10.7554/eLife.16624 . 27692068 PMC5047748

[16] Rodrigues-Campos M, Thompson BJ. The ubiquitin ligase FbxL7 regulates the Dachsous-Fat-Dachs system in Drosophila. Development. 2014;141(21):4098–103. Epub 2014/09/27. 10.1242/dev.113498 . 25256343 PMC4302899

[17] Bosch JA, Sumabat TM, Hafezi Y, Pellock BJ, Gandhi KD, Hariharan IK. The Drosophila F-box protein Fbxl7 binds to the protocadherin fat and regulates Dachs localization and Hippo signaling. eLife. 2014;3:e03383 Epub 2014/08/12. 10.7554/eLife.03383 . 25107277 PMC4144329

[18] Pan G, Feng Y, Ambegaonkar AA, Sun G, Huff M, Rauskolb C, et al Signal transduction by the Fat cytoplasmic domain. Development. 2013;140(4):831–42. 10.1242/dev.088534 . 23318637 PMC3557778

[19] Brittle AL, Repiso A, Casal J, Lawrence PA, Strutt D. Four-jointed modulates growth and planar polarity by reducing the affinity of dachsous for fat. Current biology: CB. 2010;20(9):803–10. 10.1016/j.cub.2010.03.056 . 20434337 PMC2958304

[20] Ishikawa HO, Takeuchi H, Haltiwanger RS, Irvine KD. Four-jointed is a Golgi kinase that phosphorylates a subset of cadherin domains. Science. 2008;321(5887):401–4. 10.1126/science.1158159 . 18635802 PMC2562711

[21] Simon MA, Xu A, Ishikawa HO, Irvine KD. Modulation of fat:dachsous binding by the cadherin domain kinase four-jointed. Current biology: CB. 2010;20(9):811–7. 10.1016/j.cub.2010.04.016 . 20434335 PMC2884055

[22] Matakatsu H, Blair SS. Interactions between Fat and Dachsous and the regulation of planar cell polarity in the Drosophila wing. Development. 2004;131(15):3785–94. 10.1242/dev.01254 . 15240556

[23] Ambegaonkar AA, Pan G, Mani M, Feng Y, Irvine KD. Propagation of Dachsous-Fat planar cell polarity. Current biology: CB. 2012;22(14):1302–8. 10.1016/j.cub.2012.05.049 . 22727698 PMC3418676

[24] Brittle A, Thomas C, Strutt D. Planar polarity specification through asymmetric subcellular localization of Fat and Dachsous. Current biology: CB. 2012;22(10):907–14. 10.1016/j.cub.2012.03.053 . 22503504 PMC3362735

[25] Hale R, Brittle AL, Fisher KH, Monk NA, Strutt D. Cellular interpretation of the long-range gradient of Four-jointed activity in the Drosophila wing. eLife. 2015;4 Epub 2015/02/25. 10.7554/eLife.05789 . 25707557 PMC4338440

[26] Misra JR, Irvine KD. The Hippo signaling network and its biological functions. Annual review of genetics. 2018;52:65–87. 10.1146/annurev-genet-120417-031621 . 30183404 PMC6322405

[27] Willecke M, Hamaratoglu F, Sansores-Garcia L, Tao C, Halder G. Boundaries of Dachsous Cadherin activity modulate the Hippo signaling pathway to induce cell proliferation. Proceedings of the National Academy of Sciences of the United States of America. 2008;105(39):14897–902. 10.1073/pnas.0805201105 . 18809931 PMC2567464

[28] Feng Y, Irvine KD. Fat and expanded act in parallel to regulate growth through warts. Proceedings of the National Academy of Sciences of the United States of America. 2007;104(51):20362–7. 10.1073/pnas.0706722105 . 18077345 PMC2154436

[29] Silva E, Tsatskis Y, Gardano L, Tapon N, McNeill H. The tumor-suppressor gene fat controls tissue growth upstream of expanded in the hippo signaling pathway. Current biology: CB. 2006;16(21):2081–9. Epub 2006/09/26. 10.1016/j.cub.2006.09.004 . 16996266

[30] Bennett FC, Harvey KF. Fat cadherin modulates organ size in Drosophila via the Salvador/Warts/Hippo signaling pathway. Current biology: CB. 2006;16(21):2101–10. Epub 2006/10/19. 10.1016/j.cub.2006.09.045 . 17045801

[31] Bosveld F, Bonnet I, Guirao B, Tlili S, Wang Z, Petitalot A, et al Mechanical control of morphogenesis by Fat/Dachsous/Four-jointed planar cell polarity pathway. Science. 2012;336(6082):724–7. 10.1126/science.1221071 . 22499807

[32] Rogulja D, Rauskolb C, Irvine KD. Morphogen control of wing growth through the Fat signaling pathway. Developmental cell. 2008;15(2):309–21. 10.1016/j.devcel.2008.06.003 . 18694569 PMC2613447

[33] Matakatsu H, Blair SS. The DHHC palmitoyltransferase approximated regulates Fat signaling and Dachs localization and activity. Current biology: CB. 2008;18(18):1390–5. 10.1016/j.cub.2008.07.067 . 18804377 PMC2597019

[34] Matakatsu H, Blair SS, Fehon RG. The palmitoyltransferase Approximated promotes growth via the Hippo pathway by palmitoylation of Fat. The Journal of cell biology. 2017;216(1):265–77. Epub 2016/12/30. 10.1083/jcb.201609094 . 28031421 PMC5223609

[35] Mahoney PA, Weber U, Onofrechuk P, Biessmann H, Bryant PJ, Goodman CS. The fat tumor suppressor gene in Drosophila encodes a novel member of the cadherin gene superfamily. Cell. 1991;67(5):853–68. . 1959133 10.1016/0092-8674(91)90359-7

[36] Bryant PJ, Huettner B, Held LI Jr., Ryerse J, Szidonya J. Mutations at the fat locus interfere with cell proliferation control and epithelial morphogenesis in Drosophila. Developmental biology. 1988;129(2):541–54. Epub 1988/10/01. . 3417051 10.1016/0012-1606(88)90399-5

[37] Clark HF, Brentrup D, Schneitz K, Bieber A, Goodman C, Noll M. Dachsous encodes a member of the cadherin superfamily that controls imaginal disc morphogenesis in Drosophila. Genes & development. 1995;9(12):1530–42. 10.1101/gad.9.12.1530 7601355

[38] Mao Y, Kucuk B, Irvine KD. Drosophila lowfat, a novel modulator of Fat signaling. Development. 2009;136(19):3223–33. 10.1242/dev.036152 . 19710173 PMC2739141

[39] Villano JL, Katz FN. four-jointed is required for intermediate growth in the proximal-distal axis in Drosophila. Development. 1995;121(9):2767–77. Epub 1995/09/01. . 7555705 10.1242/dev.121.9.2767

[40] Von Stetina JR, Tranguch S, Dey SK, Lee LA, Cha B, Drummond-Barbosa D. alpha-Endosulfine is a conserved protein required for oocyte meiotic maturation in Drosophila. Development. 2008;135(22):3697–706. Epub 2008/10/18. 10.1242/dev.025114 . 18927152 PMC2654389

[41] Matakatsu H, Blair SS. Separating the adhesive and signaling functions of the Fat and Dachsous protocadherins. Development. 2006;133(12):2315–24. 10.1242/dev.02401 . 16687445

[42] Ma D, Yang CH, McNeill H, Simon MA, Axelrod JD. Fidelity in planar cell polarity signalling. Nature. 2003;421(6922):543–7. 10.1038/nature01366 . 12540853

[43] Liu R, Woolner S, Johndrow JE, Metzger D, Flores A, Parkhurst SM. Sisyphus, the Drosophila myosin XV homolog, traffics within filopodia transporting key sensory and adhesion cargos. Development. 2008;135(1):53–63. Epub 2007/11/30. 10.1242/dev.011437 . 18045836

[44] Franco M, Seyfried NT, Brand AH, Peng J, Mayor U. A novel strategy to isolate ubiquitin conjugates reveals wide role for ubiquitination during neural development. Mol Cell Proteomics. 2011;10(5):M110 002188. Epub 2010/09/24. 10.1074/mcp.M110.002188 . 20861518 PMC3098581

[45] Yeh E, Zhou L, Rudzik N, Boulianne GL. Neuralized functions cell autonomously to regulate Drosophila sense organ development. The EMBO journal. 2000;19(17):4827–37. Epub 2000/09/06. 10.1093/emboj/19.17.4827 . 10970873 PMC302081

[46] Riley BE, Lougheed JC, Callaway K, Velasquez M, Brecht E, Nguyen L, et al Structure and function of Parkin E3 ubiquitin ligase reveals aspects of RING and HECT ligases. Nat Commun. 2013;4:1982 Epub 2013/06/19. 10.1038/ncomms2982 . 23770887 PMC3709503

[47] Buetow L, Huang DT. Structural insights into the catalysis and regulation of E3 ubiquitin ligases. Nature reviews Molecular cell biology. 2016;17(10):626–42. Epub 2016/08/04. 10.1038/nrm.2016.91 . 27485899 PMC6211636

[48] Greaves J, Salaun C, Fukata Y, Fukata M, Chamberlain LH. Palmitoylation and membrane interactions of the neuroprotective chaperone cysteine-string protein. The Journal of biological chemistry. 2008;283(36):25014–26. Epub 2008/07/04. 10.1074/jbc.M802140200 . 18596047 PMC2882233

[49] Lynes EM, Raturi A, Shenkman M, Ortiz Sandoval C, Yap MC, Wu J, et al Palmitoylation is the switch that assigns calnexin to quality control or ER Ca2+ signaling. Journal of cell science. 2013;126(Pt 17):3893–903. Epub 2013/07/12. 10.1242/jcs.125856 . 23843619

[50] Sun S, Reddy BV, Irvine KD. Localization of Hippo signalling complexes and Warts activation in vivo. Nat Commun. 2015;6:8402 Epub 2015/10/01. 10.1038/ncomms9402 . 26420589 PMC4598633

[51] Yin F, Yu J, Zheng Y, Chen Q, Zhang N, Pan D. Spatial organization of Hippo signaling at the plasma membrane mediated by the tumor suppressor Merlin/NF2. Cell. 2013;154(6):1342–55. 10.1016/j.cell.2013.08.025 . 24012335 PMC3835333

[52] Diamonti AJ, Guy PM, Ivanof C, Wong K, Sweeney C, Carraway KL 3rd. An RBCC protein implicated in maintenance of steady-state neuregulin receptor levels. Proceedings of the National Academy of Sciences of the United States of America. 2002;99(5):2866–71. Epub 2002/02/28. 10.1073/pnas.052709799 . 11867753 PMC122439

[53] Qiu XB, Markant SL, Yuan J, Goldberg AL. Nrdp1-mediated degradation of the gigantic IAP, BRUCE, is a novel pathway for triggering apoptosis. The EMBO journal. 2004;23(4):800–10. Epub 2004/02/07. 10.1038/sj.emboj.7600075 . 14765125 PMC380992

[54] Zhong L, Tan Y, Zhou A, Yu Q, Zhou J. RING finger ubiquitin-protein isopeptide ligase Nrdp1/FLRF regulates parkin stability and activity. The Journal of biological chemistry. 2005;280(10):9425–30. Epub 2005/01/06. 10.1074/jbc.M408955200 . 15632191

[55] Wauman J, De Ceuninck L, Vanderroost N, Lievens S, Tavernier J. RNF41 (Nrdp1) controls type 1 cytokine receptor degradation and ectodomain shedding. Journal of cell science. 2011;124(Pt 6):921–32. Epub 2011/03/08. 10.1242/jcs.078055 . 21378310 PMC3115735

[56] Wald JH, Hatakeyama J, Printsev I, Cuevas A, Fry WHD, Saldana MJ, et al Suppression of planar cell polarity signaling and migration in glioblastoma by Nrdp1-mediated Dvl polyubiquitination. Oncogene. 2017;36(36):5158–67. Epub 2017/05/10. 10.1038/onc.2017.126 . 28481871 PMC5589482

[57] Masschaele D, Wauman J, Vandemoortele G, De Sutter D, De Ceuninck L, Eyckerman S, et al High-Confidence Interactome for RNF41 Built on Multiple Orthogonal Assays. Journal of proteome research. 2018;17(4):1348–60. Epub 2018/03/22. 10.1021/acs.jproteome.7b00704 . 29560723

[58] Du J, Zhang J, He T, Li Y, Su Y, Tie F, et al Stuxnet Facilitates the Degradation of Polycomb Protein during Development. Developmental cell. 2016;37(6):507–19. Epub 2016/06/22. 10.1016/j.devcel.2016.05.013 . 27326929 PMC7365346

[59] Aicart-Ramos C, Valero RA, Rodriguez-Crespo I. Protein palmitoylation and subcellular trafficking. Biochimica et biophysica acta. 2011;1808(12):2981–94. Epub 2011/08/09. 10.1016/j.bbamem.2011.07.009 . 21819967

[60] Das Thakur M, Feng Y, Jagannathan R, Seppa MJ, Skeath JB, Longmore GD. Ajuba LIM proteins are negative regulators of the Hippo signaling pathway. Current biology: CB. 2010;20(7):657–62. Epub 2010/03/23. 10.1016/j.cub.2010.02.035 . 20303269 PMC2855397

