# Supplementary figures and images for: Early girl is a novel component of the Fat signaling pathway
Source: PLoS Genet. 2019 Jan 30;15(1):e1007955. doi: 10.1371/journal.pgen.1007955 (PMC6370246; doi:10.1371/journal.pgen.1007955)

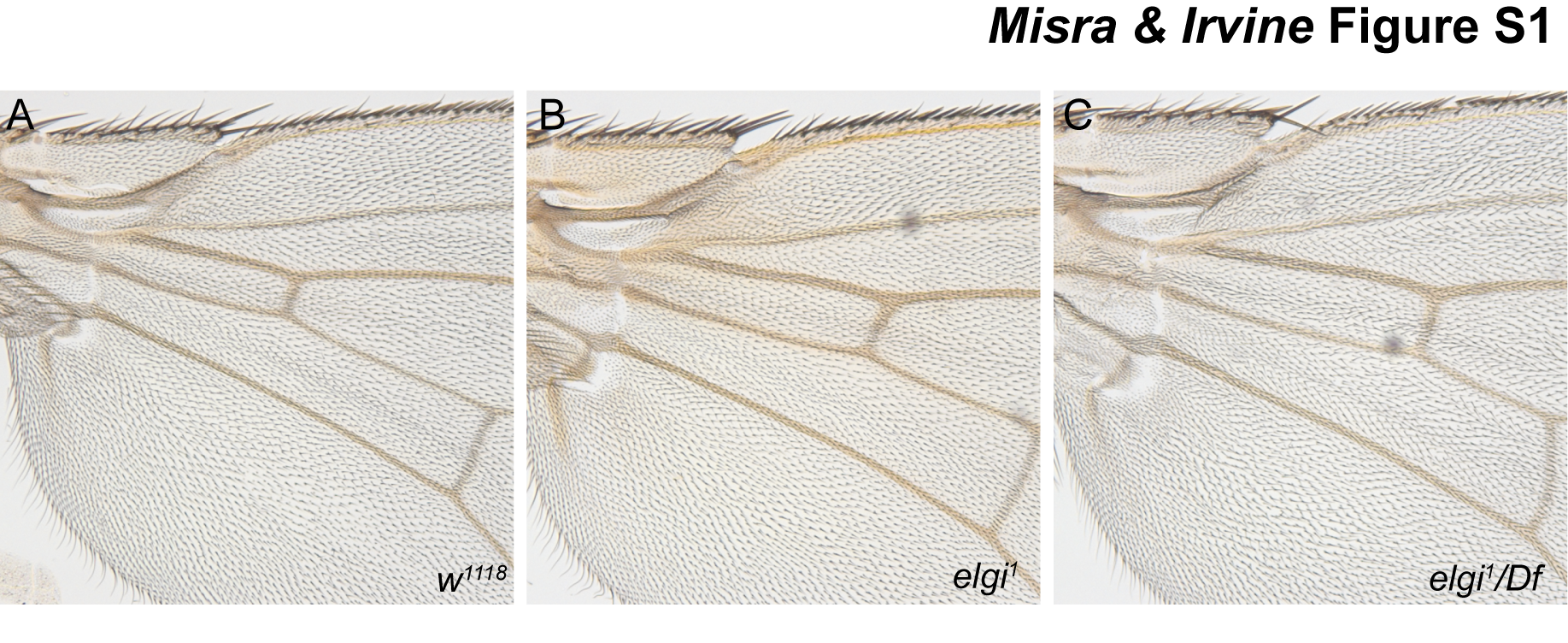

Supplement: S1 Fig — Close up of proximal wing areas showing the normal orientation of hairs from control (A), homozygous elgi1 (B), and elgi1/Df(3L)BSC575 (C) flies. (TIF) [file pgen.1007955.s001.tif]

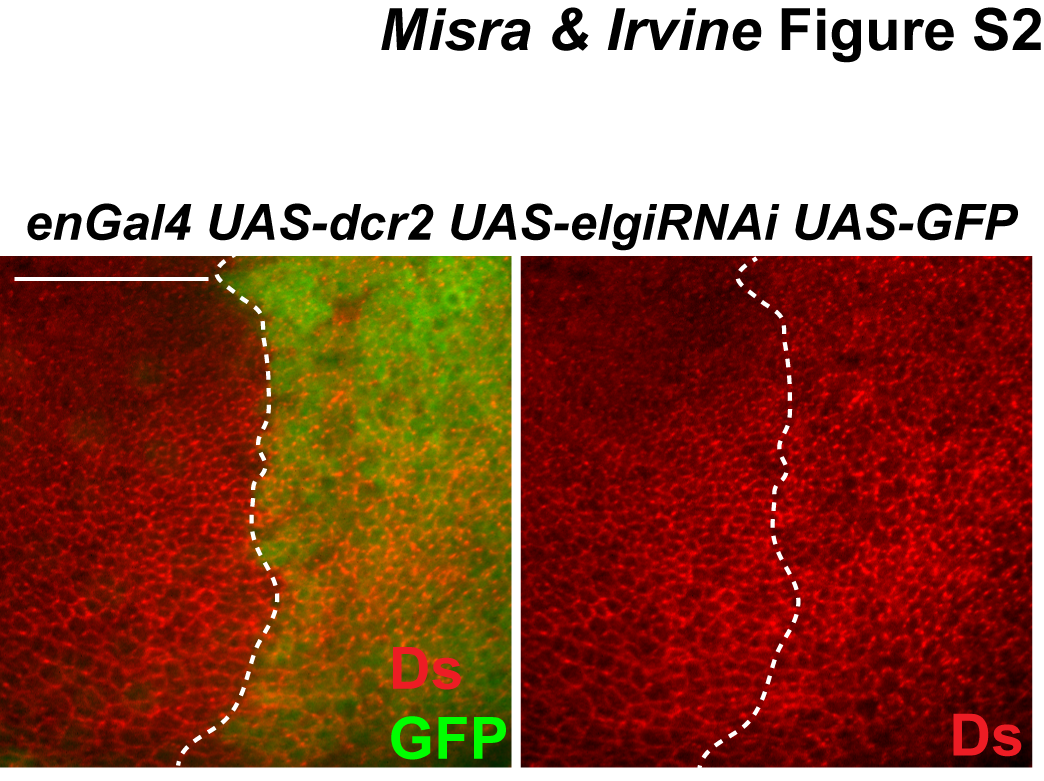

Supplement: S2 Fig — Horizontal apical sections of wing imaginal discs expressing en-Gal4 UAS-dcr2 UAS-GFP (green) UAS-elgi-RNAi and stained with anti-Ds antibody (red) showing no effect on the levels or localization of Ds in the posterior compartment (marked by GFP, green). Dashed white line marks the A-P compartment boundary. Scale bar is 33.00μm. (TIF) [file pgen.1007955.s002.tif]

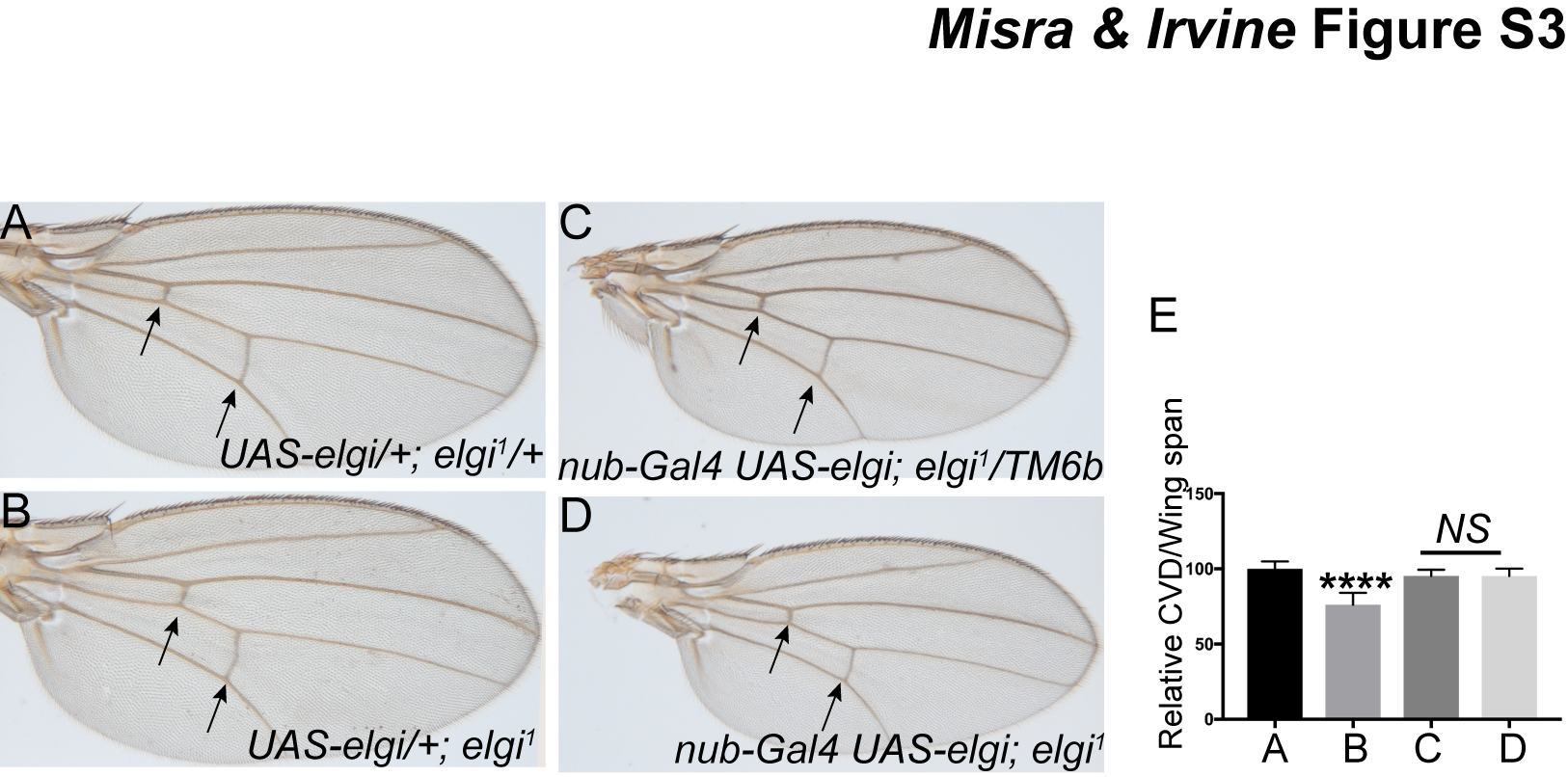

Supplement: S3 Fig — (A-D) Adult male wings from UAS-elgi-Myc-HA/+; elgi1/+ (control) (A), UAS-elgi-Myc-HA/+; elgi1 (B), nub-Gal4 UAS-elgi-Myc-HA/+; elgi1/TM6b (C) and nub-Gal4 UAS-elgi-Myc-HA/+; elgi1/TM6b (D) flies. Arrows point to the crossveins. (E) Histogram of the ratio of crossvein distance (CVD) to wing span, (normalized to the average CVD/wing span in control wings in flies of the genotypes in panels A-D, as indicated. Error bars indicate Standard deviation. **** (p<0.0001), NS not significant. (TIF) [file pgen.1007955.s003.tif]

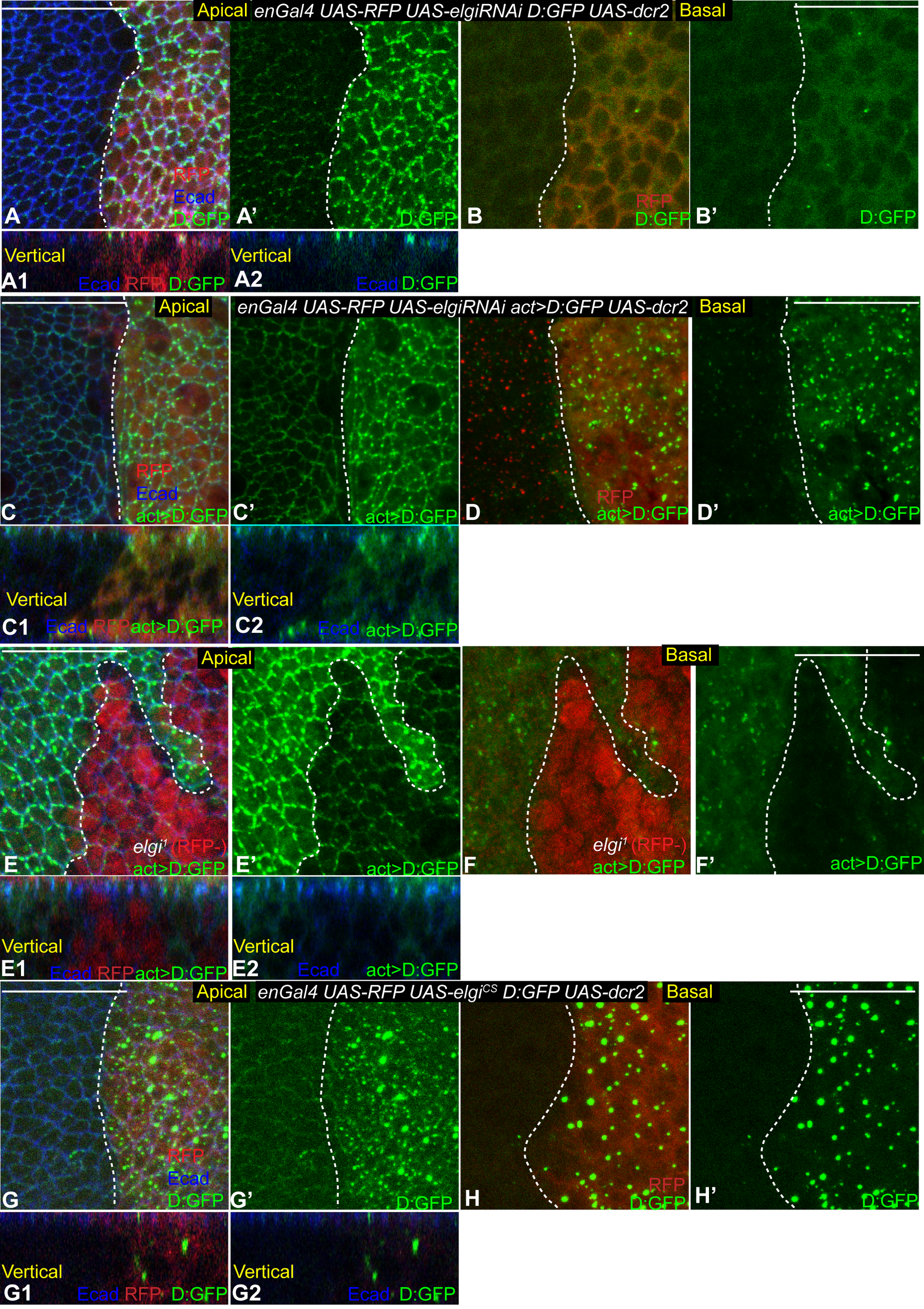

Supplement: S4 Fig — (A-D) Horizontal apical (A, A’, C, C’), basal (B, B’, D, D’), and vertical (A1, A2, C1, C2) sections of wing imaginal discs expressing en-Gal4 UAS-dcr2 UAS-RFP (red) UAS-elgi-RNAi, with either Dachs:GFP (D:GFP) (A, B) or act5C promoter driven Dachs:GFP (act>D:GFP) (C, D) showing increased levels of membrane localized Dachs:GFP (green), in the posterior compartment (marked by red). In D and D’ some punctate cytoplasmic accumulations of Dachs:GFP is seen in the basal sections. (E,F) Horizontal apical (E,E’), basal (F, F’) and vertical (E1, E2) sections of wing imaginal discs expressing Dachs:GFP under the control of the actin5C promoter (act>D:GFP) throughout, showing increased levels of membrane localized Dachs:GFP (E, E’) as well as punctate cytoplasmic accumulations of Dachs:GFP in the basal sections (F, F’) in homozygous elgi1 mutant clones, marked by absence of RFP (red). E-cad is shown in blue. (G-H) Horizontal apical (G,G’), basal (H,H’) and vertical (G1,G2) sections of wing imaginal discs expressing en-Gal4 UAS-dcr2 D:GFP UAS-RFP along with UAS-elgiCS, showing the effect on Dachs:GFP (D:GFP)(green) levels and localization in the posterior compartment (marked by RFP, red). E-cad is shown in blue. Dashed white line marks the A-P compartment boundary. Scale bar is 16.5 μm in all panels. (TIF) [file pgen.1007955.s004.tif]

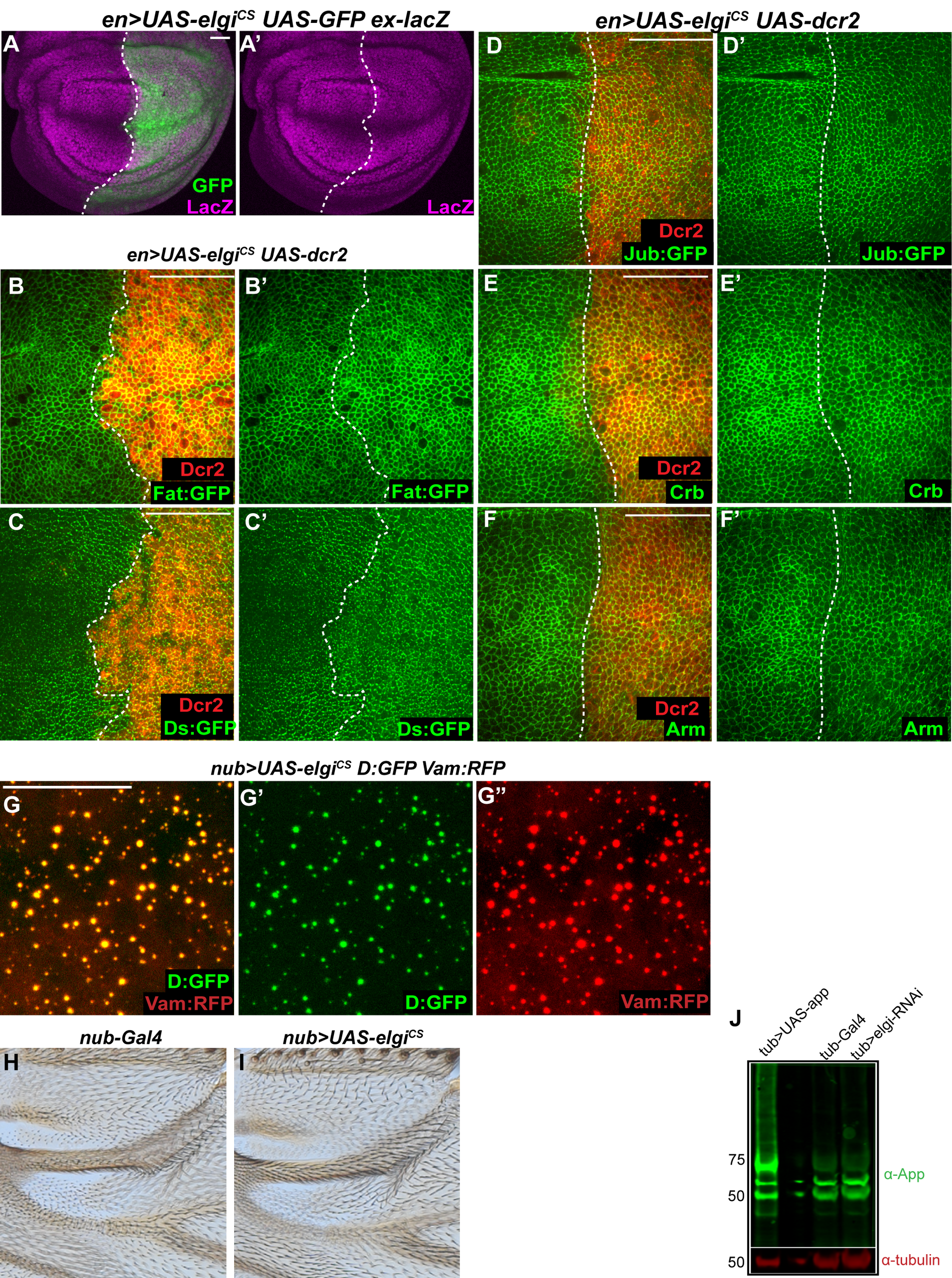

Supplement: S5 Fig — (A, A’) Third instar wing imaginal discs expressing en-Gal4 UAS-GFP ex-lacZ UAS-elgiCS stained for expression of ex-lacZ (magenta), with posterior cells marked by expression of GFP (green). Dashed white line marks the A-P compartment boundary. (B-F) Horizontal apical sections of wing imaginal discs expressing en-Gal4 UAS-dcr2 UAS-elgiCS along with Fat:GFP (B, B’), Ds:GFP (C, C’), Jub:GFP (D, D’) or stained for Crumbs (E, E’) or armadillo (Arm) (F, F’) showing that it does not affect their localization in the posterior compartment (marked by Dcr2 staining, red). Dashed white line marks the A-P compartment boundary. (G-G”) Horizontal sections of wing imaginal discs expressing nub-Gal4 UAS-elgiCS along with D:GFP and Vam:RFP showing that Vam:RFP (red) (G”) gets mislocalized with Dachs:GFP (D:GFP, green) (G’) in presence of ElgiCS. Scale bar is 33μm in A-F and 16.5 μm in G. (H, I) Close up of proximal wing areas showing the orientation of hairs from flies carrying nub-Gal4 alone (H) or in combination with UAS-elgiCS (I). (J) Western blot showing levels of App from third instar wing disc lysate from flies expressing tub-Gal4 alone or with UAS-app or UAS-elgi-RNAi. Lysates from flies expressing tub-Gal4 UAS-app serves as positive control. Tubulin was used as a control for loading and transfer. (TIF) [file pgen.1007955.s005.tif]

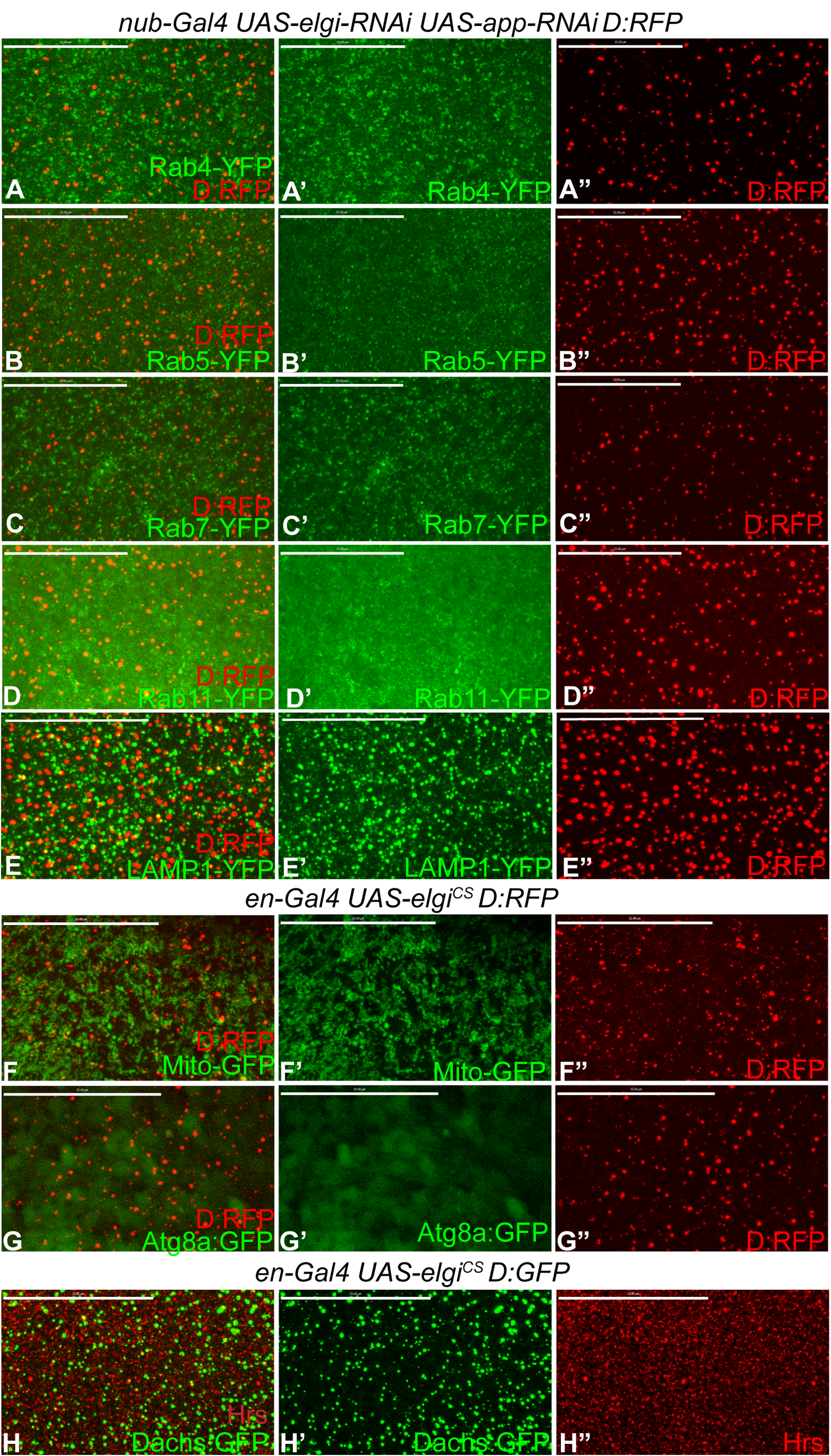

Supplement: S6 Fig — (A-E) Horizontal sections of wing imaginal discs expressing nub-Gal4 UAS-dcr2 UAS-elgi-RNAi UAS-app-RNAi UAS-D:RFP along with Rab4:YFP (green) (A, A’,A"), Rab5:YFP (green) (B, B’,B") Rab7:YFP (green) (C, C’,C"), Rab11:YFP (green) (D, D’,D") or LAMP1-YFP (green) (E,E’,E") showing that the cytoplasmic accumulations of Dachs:RFP (D:RFP, red) in absence of elgi and app do not colocalize with these markers. (F,G) Horizontal sections of wing imaginal discs expressing en-Gal4 UAS-elgiCS UAS-D:RFP (red) along with UAS-Mito-GFP (green) (F,F’,F") or UAS-atg8a-GFP (green) (G, G’,G"). (H, H’, H”) Horizontal sections of wing imaginal discs expressing en-Gal4 UAS-elgiCS, Dachs:GFP (green) stained with anti-Hrs antibody (red). Scale bar is 33 μm in all panels. (TIF) [file pgen.1007955.s006.tif]

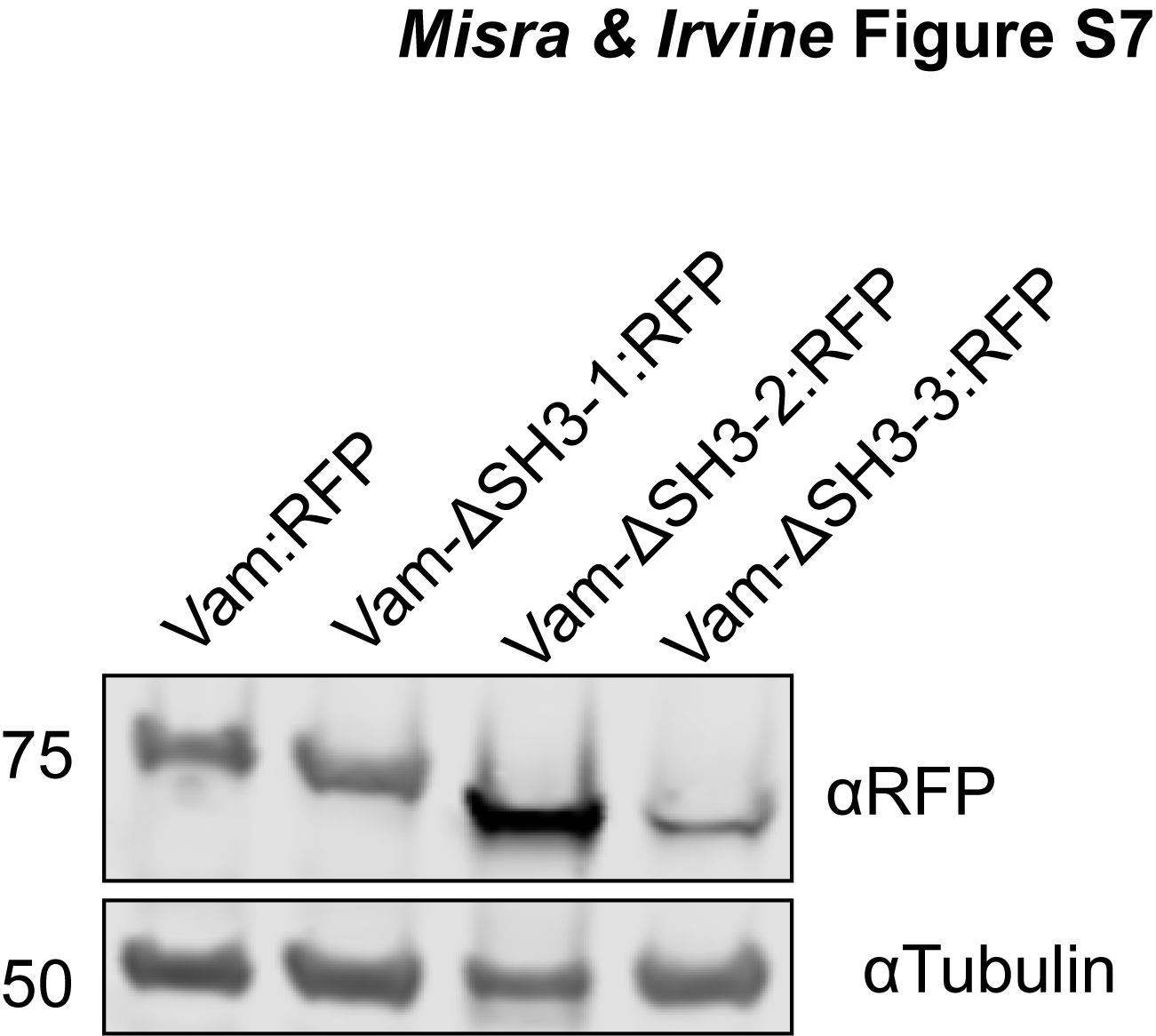

Supplement: S7 Fig — Western blot showing expression levels of full length Vam:RFP or Vam:RFP lacking the individual SH3 domains, from third instar wing disc lysate from flies expressing tub-Gal4 along with UAS-Vam:RFP, UAS-Vam-Δ-SH3-1-RFP, UAS-Vam-Δ-SH3-2-RFP or UAS-Vam-Δ-SH3-3-RFP, with all the Vam transgenes inserted at the same genomic location and detected by αRFP antibody. Tubulin was used as a control for loading and transfer. (TIF) [file pgen.1007955.s007.tif]
